# Early-Life Assets in Oldest-Old Age: Evidence From Primary Care Reform in Early Twentieth Century Sweden

**DOI:** 10.1007/s13524-018-0758-4

**Published:** 2019-01-16

**Authors:** Volha Lazuka

**Affiliations:** School of Economics and Management, Centre for Economic Demography and Department of Economic History, Lund University, Box 7083, 220 07 Lund, Sweden

**Keywords:** Early life, Primary care, Mortality, Income, Oldest-old

## Abstract

Do early-life effects of investments in public health persist to the oldest-old ages? This article answers this question by using the primary care reform in rural Sweden that between 1890 and 1917 led to the establishment of local health districts, together with openings of hospitals and recruitments of medical personnel, as a natural experiment in early-life environmental conditions. The initiatives undertaken within these districts targeted control of infectious diseases, including various isolation and disinfection measures. This study applies a difference-in-differences method combined with propensity score matching to register-based individual-level data for Sweden from 1968 to 2012 and to multisource, purposely collected data on the reform implementation. Providing pioneering evidence for such a distal relationship (ages 78–95), this study finds that treatment through primary care in the year of birth leads to a significant reduction in all-cause mortality (4 % to 6%) and mortality from cardiovascular diseases (5 % to 6 %) and to an increase in average incomes (2 % to 3 %). The effects are universal and somewhat stronger among individuals from poor socioeconomic backgrounds and at higher baseline levels of disease burden.

**Electronic supplementary material:**

The online version of this article (10.1007/s13524-018-0758-4) contains supplementary material, which is available to authorized users.

## Introduction and Previous Research

The oldest-old (80+) represent the fastest growing age group in developed countries and are projected to account for one in five older persons by 2050 globally (United Nations 2015). Undoubtedly, societies with healthy elderly are better able to overcome challenges related to rising costs in health and social care (Bengtsson 2010; Wise 2010). For these reasons, identifying the factors that lead to long-term survival and prosperity is increasingly important. Originating from Barker (1991, 1994), the epidemiological literature has developed the concepts of developmental plasticity and critical periods linking particular environments and events in early life to the development of chronic diseases as a person ages. For the historical cohorts, Fogel (1994) and Fogel and Costa (1997) advocated the importance of nutrition of fetuses and newborns for health and productivity in adulthood. Finch and Crimmins (2004) proposed that decreased exposure to infectious diseases and inflammation during early life, reflected in *cohort morbidity phenotype*, stands behind such distal associations. A response by Barbi and Vaupel (2005) argued that cohort effects are modest compared with period effects, more recently coming from medical advancements benefiting the elderly. What is left reconciled in this debate is that the earlier cohorts should experience greater contributions from the cohort factors influencing increases in life expectancy. If so, as Fogel (1994) argued, huge social investments around the turn of the twentieth century—under focus in this study—should inevitably yield large payoffs via cohort mechanisms through the end of the century.

Studying the consequences of early-life beneficial treatments in the (very) long term is challenging because survivors tend to be negatively selected (Bozzoli et al. 2009; Zajacova and Burgard 2013). This selection accumulates with age, implying that for the elderly, early-life effects should be large enough to be observable. Recent economic and historical studies, based on quasi-experimental designs, showed positive effects resulting from inputs into early-life health primarily among individuals at adult ages (Almond and Currie 2011; Currie and Rossin-Slater 2015). Relying on negative shocks to early-life health, this literature has found effects on adult heath and cognition from exposure to hunger episodes *in utero* (e.g., Almond et al. 2010; Lindeboom et al. 2010; Stein et al. 2007) and business cycles in year of birth (e.g., van den Berg et al. 2010, 2011). Micro-level studies have consistently demonstrated that in societies with substantial infectious disease prevalence, disease outbreaks in year of birth elevate adult and old-age mortality (e.g., Bengtsson and Lindström 2003; Costa 2000; Schellekens and van Poppel 2016) and dampen cognition and socioeconomic outcomes (e.g., Bengtsson and Broström 2009; Case and Paxson 2009; Myrskylä et al. 2013). Appealing to the importance of policy treatments, several studies have found positive effects of water purification or medical innovations in the first half of the twentieth century on adult earnings among individuals affected at birth and in early childhood (Beach et al. 2016; Bhalotra and Venkataramani 2011; Glied and Neidell 2010; Hjort et al. 2017; Lazuka 2018a). Evidence on the very long-term effects of early-life programs is extremely limited. As one exception, Aizer et al. (2016) studied the impact of the provision of cash transfers to poor families in the United States in 1911–1930, when public health interventions were lacking, and showed that it affected treated children by improving incomes in their adulthood and survival to their 60s to 90s. Albeit for the younger ages, Bhalotra et al. (2017) found that infant care programs—primarily, provision of preventive care—in 1931–1933 in Sweden enhanced survival to their 40s to 70s.

Early-life health programs targeting rural areas have been found to be particularly important for later-life outcomes. For the rural United States, eradication of hookworm disease (circa 1910) (Bleakley 2007) and the anti-malaria campaign (circa 1920) (Bleakley 2010) before age 18 led to substantially higher wages in adulthood relative to low-infection regions and earlier-born cohorts. More abundant literature examining the effects of early-life health policy in rural areas is limited to the settings of developing countries and short time spans (Currie and Vogl 2013; Strauss and Thomas 2008). For instance, a follow-up of a randomized experiment in rural Guatemala conducted in the 1960s–1970s to provide nutrition, curative care, and immunizations showed that children treated in the first two years of life had increased wages as young adults (Hoddinott et al. 2008). Baird et al. (2016) found positive effects of a deworming experiment in childhood in the late 1990s in rural Kenya on self-reported health and number of hours worked. A quasi-experimental study of the impacts of maternal and child health program for rural Bangladesh implemented in the 1970s–1980s found positive long-term effects on adult height (Barham et al. 2016). The impacts of conditional cash transfer programs implemented in rural areas, which included early-life health care components, are ambiguous for the medium term and likely have not been realized yet (Parker et al. 2008).

Considerable epidemiological evidence suggests that environmental factors in early life influence morbidity and mortality later in life. Among the first, Barker et al. (1989) and Barker (1994) documented strong associations between low birth weight and exposure to infectious diseases in infancy and old-age chronic mortality for cohorts born in 1911–1925 in England and Wales. Only the earlier work by Barker was explicit about the importance of early infections for later-life mortality, whereas his later work focused entirely on the role of maternal nutrition. Still, epidemiological research has consistently found correlations between markers of inflammation, especially in the early neonatal period, originating from adverse exposures in early life, as well as incident cardiovascular disease, metabolic syndrome, later-life disability, and all-cause mortality (Lefkou et al. 2006; McDade et al. 2010). Finch and Crimmins (2004) and Finch (2007) reviewed the relevant epidemiological studies and argued that the reduction in chronic mortality among the elderly throughout the twentieth century is fully linked to cohort improvements in infectious disease burden because all chronic diseases related to aging—especially cardiovascular and metabolic diseases—have inflammatory components. Exposure to respiratory infections in the first year of life leads to underdevelopment of lungs and to lung damage, which persists throughout life and manifests in chronic respiratory diseases (Bengtsson and Lindström 2003). A characteristic of infections is their ability to disseminate into the blood and arterial walls and, in some cases, to persist in tissues for many years given that the insult occurs in early life, causing chronic inflammation (Liuba and Pesonen 2005). Prevention from adverse environmental exposures could have lasting health effects, and direct inputs in terms of primary and supportive care in infancy could also critically influence the development of stress response and may alter the long-term inflammatory process (Danese and McEwen 2012; Miller et al. 2011). All beneficial environmental factors acting in early life can affect almost any aspect of the immune system, leading to a decreased later-life risk of cardiovascular disease and diabetes (Leifer and Dietert 2011).

The current study contributes to the literature in several ways. Studies on the role of public health investments at the turn of the twentieth century in the outcomes at old and oldest-old ages are scarce. In rural Sweden, these investments primarily targeted the establishment of primary care facilities around health districts (Medicinalstyrelsen 1907). This study’s most important contribution is that it provides pioneering evidence for the long-term effects of such population-based reform for individuals treated in early childhood and observed at the oldest-old ages. In the current study, I focus on both health and income outcomes—rarely investigated simultaneously—enabling me to relate early-life health investments to later-life health and then to income, and to calculate a social rate of return to these investments, which emerges as an additional contribution. The closer look at the cause-specific mortality taken in this study could also point to the determinants of chronic diseases and conditions that today constitute the main burden of aging societies (Wise 2010). Moreover, this study also contributes to investigations of the impacts of an early-life health policy targeted at residents of rural localities, providing new evidence for the existence of such distal impacts in the European setting. The initiatives undertaken with health district reform in Sweden at the turn of the twentieth century resemble the experience of all developed countries in the past and of developing countries more recently. The reform in Sweden focused on the prevention of infectious diseases under conditions of new bacteriological regulations through isolation and disinfection. In addition to exploring the whole lifespan under analysis, this study points to the long-term importance of these public health components. During period under study, no drugs against diseases or efficient vaccines were available, and no food control, housing, or water supply treatments launched in parallel (Niemi 2007).

Based on a quasi-experimental design, this study aims to explore the effects of Sweden’s establishment of primary care facilities in 1890–1917 made in early childhood on health and pension and capital incomes between ages 78 and 95. Throughout the twentieth century, Sweden not only was a longevity frontrunner—exceeding 80 years already in the 2000s (Colchero et al. 2016)—but also experienced an unprecedented increase in real income, measured (for instance) at 2 % in per capita terms (Schön and Krantz 2012). Comparing cohorts before and after the public health investments can be misleading because some share of the differences may relate to secular mortality decline. To control for this, I apply a difference-in-differences (DiD) approach, which compares differences in the outcomes across cohorts born in the treated parishes with differences across the same cohorts born in control parishes. The gradual implementation of the reform across parishes and cohorts, together with a rich supplementary data set on the characteristics of the parishes and health districts, enables estimation of the DiD, attributed to the presumably causal effect of the reform. I find that due to the reform, individuals treated in the year of birth attained decreases in all-cause mortality risk at approximately 4.1 % to 6.0 %, roughly equivalent to 0.5–0.7 additional years spent alive. The positive effect of the reform on total income amounts to 1.6 % to 2.5 %.

## 1890 Health District Reform

From 1890 until the 1920s, all rural communities in Sweden, constituting three-fourths of the total population, gradually received access to public health care in the form of local health districts (see online appendix A for additional contextual information). Dating back to 1773, the provincial doctor district (*provinsialläkardistrikt*) was organized around an assigned doctor, midwives, and a hospital. Until the mid-nineteenth century, the number of centrally introduced health districts amounted to 2 per 100,000 inhabitants and disproportionally covered the more urbanized locations (Medicinalstyrelsen 1907). The local health administration instructions, introduced in the 1870s, prescribed each parish or group of parishes to set up a local public committee to address health matters, in particular by controlling the spread of infectious diseases (Lindblom 1967). The location-initiated creation of health districts accelerated accordingly, beginning in the 1880s, albeit initially sluggish and favoring wealthy and industrialized locations (Medicinalstyrelsen 1907). Driven by international achievements in municipal governments and medicine as well as by rapid industrial and population growth in the countryside, Swedish state authorities in 1890 announced a reform aimed at creating medical districts in all parts of the country, giving this opportunity equally to economically disadvantaged areas. According to the reform, each group of parishes with 8,000–12,000 inhabitants applying for a public health district could be subsidized with 1,500 SEK from the government and had to accumulate 2,500 SEK from local sources. Primary care was provided strictly within the boundaries of the health districts based on the contracts signed at the community level (Engberg 2007). Additionally, the state began to stimulate the graduation of young medical professionals and attract them to rural parishes, primarily by guaranteeing career promotions and public pensions. The reform, therefore, was designed centrally to bring access to public health care, with more local resources devoted to it for rural populations.

**Fig. 1:**
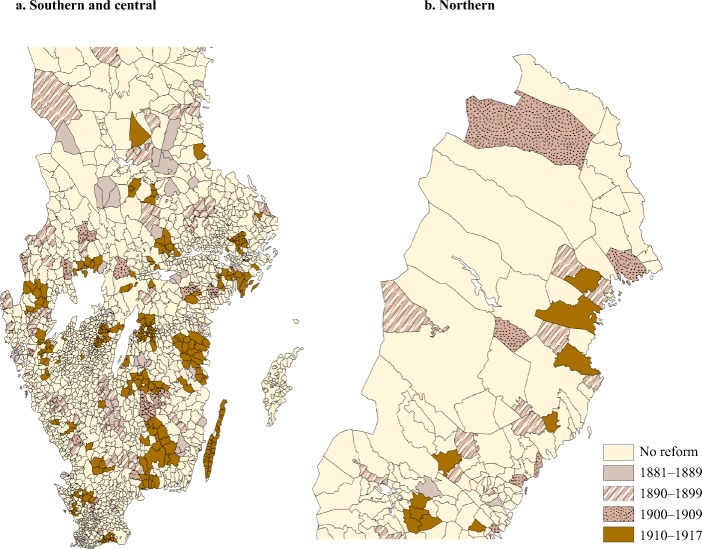
Implementation of the 1890 health district reform in Sweden. Parishes that implemented reform in 1890–1917 are included in the implemented sample. “No reform” denotes parishes that did not implement the reform in 1881–1917 and are included in the matching procedure. *Source*: Historical GIS maps from the Swedish National Archives (Riksarkivet 1890–1917) merged with data on reform dates (online appendix B).

Figure 1 demonstrates the progress in health care reform across Sweden and the considerable variation in timing across different regions. Data purposely collected for this study (see online appendix B for data sources) show that separate health districts were introduced gradually throughout rural parts of Sweden, adding 124 districts to the 163 districts that existed prior to the expansion; 17 of them were created in 1881–1889, and 107 districts, funded by the subsidies, were created in 1890–1917. My calculations show that the objectives of the reform were realized (online appendix C). The average health district cost the public approximately 3,100 SEK in real terms, which was spent primarily on the opening of a hospital or a medical station, and the employment of doctors and midwives. Access to health care at the community level was given to 12,000 inhabitants who resided in six to seven parishes, on average. At a minimum, the 1890 reform doubled the rural population’s access to public health care. Induced by the reform, public health spending increased by more than 680 SEK per 1,000 inhabitants in real terms, and parishes additionally employed 6 midwives and 2 doctors per 10,000 inhabitants. These figures also exceed the baseline rates of health care developments in the countryside as a whole: investment in health care increased by only 270 SEK per 1,000 inhabitants, and 1 midwife and 0.6 doctors per 10,000 were employed additionally there (Statistiska Centralbyrån 1880–1917).

Taking advantage of the plentiful, purposely built parish-level data set, I analyze which factors were associated with the implementation of the reform. If existent, the influence of correlations between the reform initiation and observable and unobservable factors on the results should be fully controlled by the method discussed later. Perhaps the forerunning parishes had other favorable characteristics, such as wealth or health, that fostered the beneficial long-term effects rather than the reform. Instead, as can be seen with regard to wealth in terms of real total investment or the fraction of the active population in the labor force prior to the reform (Table 1), the parishes that established the reform earlier were likely to be, on average, poorer compared with later-adopting parishes. Regarding population health, the reform under analysis was introduced independently of the disease conditions in the parishes, as measured, for example, by the share of the disabled or the mortality rate under age 15. These indications are similar to using a nonlinear specification of a year of establishment, following the observation that the number of parishes that established a health district increased over the study period. Improved access to health care was likely to be driven by the nationwide fear of epidemics and the recognition of the lack of local competence in their prevention (Lazuka et al. 2016). No other health measures—such as water supply and sewerage installations, sanitation measures, food inspections, and improvements in housing conditions—occurred in parallel with the primary care measures.

**Table 1:** Factors of staggered implementation of the reform, 1890–1917: OLS regression estimates

	Year of Implementation			
	Levels: 1880		Differences: 1890 to 1880	
	Mean (SD)	Estimate (*p* value)	Mean (SD)	Estimate (*p* value)
Log Real Investment in Health Care per Parish	7.260	3.994**	––	––
	(0.796)	(.000)		
Log Real Education, Infrastructure, and Welfare Spending per Parish	9.402	3.002**	––	––
	(0.675)	(.000)		
Log Population per Parish	7.232	–1.188*	–0.027	–12.510**
	(0.753)	(.016)	(0.123)	(.000)
Share of Elite and Industrial Workers in Population of Men Aged	0.266	4.984	–0.009	–3.140
	(0.109)	(.146)	(0.079)	(.509)
Share of Agricultural Workers in Population of Men Aged 15–55	0.406	1.146	0.013	8.072^†^
	(0.103)	(.751)	(0.088)	(.056)
Mean Age of Woman	29.250	0.196	1.319	1.021
	(1.890)	(.322)	(1.418)	(.834)
Share of Women in Total	0.507	20.445	0.001	0.234
	(0.017)	(.341)	(0.015)	(.373)
Share in Labor Force in Total, Ages 15–55	0.666	9.058*	0.028	32.367
	(0.095)	(.021)	(0.077)	(.196)
Share Married in Total, Ages 15–55	0.480	–8.853	0.002	4.546
	(0.055)	(.189)	(0.037)	(.656)
Mean Family Size	4.015	–2.987**	–0.189	–1.105
	(0.449)	(.000)	(0.305)	(.366)
Share Under Age 1 in Total	0.025	–93.097	–0.002	–48.321
	(0.005)	(.189)	(0.006)	(.410)
Share Above Age 55 in Total	0.150	15.377	0.028	19.750
	(0.024)	(.314)	(0.021)	(.270)
Mortality Rate Under Age 15	0.753	–0.074	6.226	–0.000
	(2.642)	(.600)	(18.157)	(.983)
Share of (Non)disabled	0.992	25.982	0.000	78.553
	(0.006)	(.697)	(0.006)	(.234)
Railway	0.234	0.703	–0.077	0.364
	(0.424)	(.425)	(0.447)	(.664)
Water Supplies Improvements	0.037	3.735^†^	0.109	2.548
	(0.188)	(.060)	(0.055)	(.251)
Number of Parishes	492	492	492	492

*Notes*: All characteristics are at the parish level. The dependent variable is year of implementation (continuous, mean = 1,906.957, SD = 8.263). Each coefficient is estimated separately. Means are displayed for the respective independent variables, with standard deviations below them in parentheses. See online appendix B for data sources and descriptions. Parish-level indicators are gathered from censuses 1880, 1890, 1900, and 1910, and from Statistiska Centralbyrån, BISOS U and K 1880–1917. Public investment data (*Log Real Investment in Health Care per Parish* and *Log Real Education, Infrastructure, and Welfare Spending per Parish*) are gathered for the year 1880 and reform years (varying across 1890–1917). Because of the upward trend in all types of investments, differences between these investments correlate with a year of implementation by construction; therefore, the results for these covariates are omitted.

^†^*p* < .10; *p < .05; **p < .01

Studying yearly reports of provincial doctors in treated and untreated districts (Medical History Database 1885–1900; Riksarkivet 1885–1914) shows that the initiatives undertaken in the parishes as a result of the 1890 reform were intended to prevent the spread of infectious diseases (see online appendix A for more detail). Because of the bacteriological discoveries in the 1870s–1880s, the public acquired such tools and could apply them under the guidance of the newly graduated medical personnel, although no cures or vaccines were available until the late 1930s. In response to the cholera epidemic, obligatory isolation of the infected residents or travelers was in place in the populous countryside beginning in the early 1890s (Svensk Författningssamling 1893). The cottage hospitals or health stations in the parishes were built for this reason, whereas chronic patients for many years were transported to neighboring cities for in-patient care. As a result of the reform, the newly assigned provincial doctors intensified disease tracing, monitoring, isolation of infected persons, and disinfection of belongings and rooms with disinfectant apparatuses (Engberg 2007). A new initiative undertaken during the reform was encouragement and realization of school closures during the outbreaks of scarlet fever, measles, diphtheria, and whooping cough. Previously, midwives by themselves could be carriers of disease, but under the control of medical doctors and with the introduction of the disinfection instructions for childbirth in 1881, the organization of health districts encouraged the employment of new graduates from midwifery schools who were competent in modern medical knowledge and the use of antiseptics (Lazuka 2018b). Additionally, the medical practitioners brought surveillance and relief from disease. Beneficial treatment by the reform did not correlate with socioeconomic characteristics of the parishes, which was expected given that health care was provided to the public through redistribution at no or negligible cost to the recipients (Curtis 2011).

## Data

### Administrative Individual-Level Data for the Outcomes

The individual-level data used in this study come from Swedish administrative registers. I use the Swedish Interdisciplinary Panel (SIP), which combines the multiple administrative registers for all individuals residing in Sweden from 1968 until 2012 and having family links after 1930, tracked through unique personal identifiers (see online appendix D). The SIP contains information on the individual’s county and parish of birth, which have been merged with the data on health districts. In the period under analysis, the parish of birth is accurate and corresponds with the location of the mother’s residence (Riksskatteverket 1989). To ensure consistency in ages for the cohorts born between 1890 and 1917, I focus on outcomes at ages 78–95. Of first-year survivors of these cohorts, 38.8 % are observed to reach this age. In the Robustness Analyses section, I further analyze the cohorts with regard to the presence of various selection features. The population and death registers provide records for the individual’s time at risk and the date of death. In addition, the cause-of-death register gives the primary cause of death. Guided by the epidemiological literature, I further classify the primary cause into six groups: infectious/respiratory diseases, cardiovascular diseases, cancer, diabetes, degenerative diseases, and other causes (see online appendix E).

I obtain data on income from the income and taxation register. In the observation period, all individuals, with the exception of those in several earlier cohorts, were under the pension scheme introduced in 1960 that could be claimed beginning at age 67. The scheme provided a flat basic rate with a supplementary benefit determined as a percentage of the average 15 highest paid years (Kruse 2010). Although ceilings in the payments existed, the pensions were higher with either a longer working period or a steeper earnings profile. The pension system also contained a widow’s pension, which could be paid either until the death of the widow or remarriage; similar benefits could be accrued for men. Because I want to avoid changes in the registration of different types of income occurring during the period under study and because pension could be capitalized, I use total income, which includes pension (90.7 %) and capital income (9.3 %) for the age groups under study. For those individuals for whom both a base pension and a contribution dependent on labor productivity are known (from 1991), the latter constitutes 43.7 % of the final income measure (calculated from the SIP). I rely on the arithmetic mean of the real annual income between age 78 and the year before death or age 95 as the preferred measure of long-term income. To provide a comparison between the cohorts with different earnings profiles and to impede their disproportionate influence on the results, I use the logarithm of this measure as an outcome in the models.

### Parish-of-Birth Data on the Reform and Other Characteristics

Given the rollout of the health district reform, accurate data for the division of the parishes into health districts and its changes are required (see online appendix B for a detailed description). I collect this information from several sources. Primarily, the reports of the national health board on provincial doctor districts contain detailed data on the allocation of the parishes as well as the creation dates and funding of the new districts (Medicinalstyrelsen 1907, 1939). Because the passage of the establishment acts may be misleading about the timing of movements in actual policy (Engberg 2007), I verify these divisions with several archival and statistical sources. These sources provide information on the number of the medical personnel employed (doctors and midwives), as well as public spending on health care and on education, infrastructure, and welfare. In case of investment, I gather public investment series for each parish before and after the establishment year and aggregate them to a health district level, which allows me to carefully determine the intervention dates. The estimation sample includes rural (*land*) parishes only. I exclude from the analysis the rural parishes that developed through the period into market cities (*köping* or *stad*) in order to avoid the overlap with water purification interventions, and I exclude parishes that experienced several health district reallocations or that had uncertain adoption dates (220 of 2,353 parishes). I stop following the establishment of medical districts in 1917, mainly because of the reorganizational character of the reform afterward.

In the individual administrative data, cohorts from 1890–1917 are not linked to their families of origin. To fill the gap in the individual’s background characteristics, which is highlighted as necessary in early-life studies (Kuh and Ben-Shlomo 2007), I augment abundant parish-level information from other national records. The Swedish decennial censuses of 1880–1910 are the main sources (Riksarkivet 2014). The counts contain the occupation names, their historical international classification of occupations (HISCO), and status codes, which I further standardize into a historical international social class scheme and obtain a measure of socioeconomic status consistent between the cohorts (HISCLASS; van Leeuwen and Maas 2011). I construct various socioeconomic and demographic variables at the parish level, such as the share of elite and industrial workers, whether the parish had a railway, the mean family size, and the size of the population among others (see online appendix B for a full set). Introducing population density, where area measures are calculated based on the historical maps (Riksarkivet 1890–1917), instead of the logarithm of the population provides similar results. I complement this group of variables with information on deaths under age 15 gathered from the national death register (Sveriges Släktforskarförbund 2017).

## Methods

The establishment of primary care facilities around new health districts can be considered as an improvement in the individual’s early-life environmental conditions. Following the previous literature and in light of the prime focus of the reform on the reduction of exposure to infectious disease, I merge the year of the reform implementation to the year of birth. Given the gradual implementation of the reform throughout parishes in Sweden in 1890–1917, I apply a DiD approach in the following form:

1 $$ {y}_{ipb}=\upalpha +\upbeta {Post}_b\times New\ {Health\ District}_p+{\upeta}_p+{\uplambda}_b+{\boldsymbol{\upvarphi}}_i+{\upvarepsilon}_{\mathrm{i} pb}, $$

where *y*_*ipb*_ denotes the outcomes for individual *i* born in parish *p* in the birth year *b*; *Post*_*b*_*× New Health District*_*p*_ is an indicator for the new health district established in parish *p* in a year of birth *b* and remained in place in the post-treatment period; η_*p*_ denotes parish-of-birth fixed effects; λ_*b*_ denotes year-of-birth fixed effects; and **φ**_*i*_ are individual-specific characteristics (sex).

Equation (1) can be estimated for the income outcome by means of a linear fixed-effects model, but I apply a Cox proportional hazard model for mortality to capture its nonlinearity (Cox 1972). In doing so, to avoid the possible bias introduced by estimating a large number of incidental parameters for the parishes of birth, I implement a DiD method by using the extension of the model, a stratified partial likelihood model:

2 $$ {h}_{ipb}(a)={h}_p(a)\times \exp \left(\upbeta {Post}_b\times New\ {Health\ District}_p+{\uplambda}_b+{\boldsymbol{\upvarphi}}_i\right), $$

where *h*_*ipb*_(*a*) denotes the all-cause or cause-specific hazard of death for individual *i* born in parish *p* in the birth year *b* at age *a*; and *h*_*p*_(*a*) denotes a set of baseline hazards separate for each parish-of-birth *p* (strata) that get absorbed into the unspecified function of age. Equation (2) thus eliminates the effects for the parishes of birth from the likelihood function, in analogy with parish-of-birth fixed effects (Ridder and Tunali 1999). Duration models adjust for left-truncation at age 78. In all models, to account for the location-level unobserved correlation, I cluster standard errors by the parish of birth.

The parishes that implemented the reform between 1890 and 1917 could be distinguished from those that did not implement the reform in this period, with the latter being either (1) located closer to the medical centers, existing prior to 1880, and having substantially larger public health inputs; or (2) established provincial districts much later, after 1917, until the completion of the reform in the mid-1940s. To avoid comparing such dissimilar groups, I include in the baseline sample parishes that established a provincial doctor district at some point in time between 1890 and 1917 (*Implemented* sample). Of 2,133 rural parishes, 492 are included into analysis, and the representatives of 414 are eventually observed in the individual data, with no systematic differences compared with those born in the unobserved parishes (see online appendix D). The state guaranteed the placement of a provincial doctor for at least five years, and the majority of the districts had a longer duration (Medicinalstyrelsen 1907); I therefore consider pre- and post-treatment periods to be five years each. Given this strategy, I expect to find beneficial effects, captured with β*,* for those treated by the reform in the year of birth compared with those who were aged 1–5 at the time of the reform implementation and living in the same parish (because each parish in this sample eventually established a new health district). Comparing the treated groups with those beyond childhood is not possible because of data limitations.

Because the empirical strategy relies on the random nature of the timing of establishment of health districts and parallel developments in the outcomes across the parishes, I use several approaches to address concerns about their retention. The treated parishes are located in different parts of the country, so to eliminate the treatment effect from any secular trends at the level of the county of birth, I introduce interactions between the county-of-birth dummy variables and year-of-birth linear trends in the first approach. Second, based on a multisource parish-level data set described earlier and following Hoynes et al. (2016), I control for trends in the observable pre-treatment characteristics by including interactions between the parish-of-birth characteristics, such as levels in 1880 and their changes from 1880 to 1890, and cohort dummy variables. This should balance the treated and untreated groups based on observable pre-treatment characteristics, which are deemed important because the timing of the reform correlated with certain parish-of-birth characteristics. In using a DiD method, one should be especially concerned that the outcomes for the treated and untreated groups before the reform implementation exhibited parallel trends. Any unobservable factors at the parish-of-birth level could determine the development of the outcomes instead of the reform. I first investigate this with an event study design, by looking at the treatment effects by each cohort before and after the reform implementation. Additionally, I introduce parish-specific linear time trends; inclusion of quadratic trends instead produces similar results.

The reform did not affect all rural parishes in Sweden; the majority of them enjoyed the health system that existed prior to 1890. As a final approach, following Hjort et al. (2017), I match each treated parish of birth with one that remained untreated, based on the pre-1890 parish-of-birth and health district characteristics, thereby achieving symmetry in the pre-treatment trends (see online appendix F for details). These characteristics follow a standard model of public health care utilization (e.g., Kifmann 2005). For the matching approach, I calculate propensity scores and apply nearest neighbor matching, in which I allow only one control without replacement and impose a common support restriction. Using the constructed sample with individuals born in 660 parishes (*Matched* sample), I assign implementation dates to the matched parishes based on their treated counterparts and estimate the coefficients in Eqs. (1) and (2). Therefore, this strategy compares the long-term outcomes of the individuals actually treated in the year of birth with those born in the same year and in a similar parish that was not treated; net of parish-specific heterogeneity, this difference is captured by parameter β. Based on this sample, I can estimate the effects for the treatment beyond year of birth, *in utero* or at older ages in early childhood, which do not appear to be different from null (see online appendix F). I also gather the data on the recruitments of doctors and midwives across the implemented and matched parishes (not related to the establishment of the districts), and controlling for them does not affect the main results.

Table 2 presents descriptive statistics for the estimation samples. Conditional on survival to age 78, individuals died at a mean age of 86–87. This characteristic of samples does not deviate from the actual life expectancy at age 78 for the same cohorts in Sweden, which is equal to 8.5 years (Human Mortality Database 2017; see also online appendix D). More than 90 % of individuals died between ages 78 and 95, with the largest fraction of deaths due to cardiovascular diseases (57 %), followed by cancer (16 %), degenerative diseases (13 %), infectious/respiratory diseases (9 %), diabetes (2 %), and other causes (3 %). The share of men in the sample is approximately 45 %, which is expected given their lower survival to old age compared with women. The logarithm of the average income in the main estimation sample is 7.7 units, which aligns well with the same cohort measure for the total population (calculated from the SIP). In online appendix G, I compare the age group under analysis (ages 78–95) with the same cohorts in younger age groups observed in registers (ages 50–66 and 67–77). The individuals under study should be viewed as similar to those at adult ages in terms of cardiovascular diseases as a primary cause of death, primary education as the mean level of education, and agriculture as the main sector of employment. As expected, proportions of individuals who lost a partner or died are substantially higher after age 78.

**Table 2:** Descriptive statistics of the estimation samples: Means, with standard deviations in parentheses

	Implemented (*I*) Sample	Matched (*M*) Sample
Mortality Sample, Ages 78–95		
*Post*_*b*_ × *New Health District*_*p*_	0.540	0.254
	(0.498)	(0.435)
Mean death age (noncensored)	85.85	85.81
	(4.799)	(4.805)
Mean censoring age	86.55	86.50
	(5.220)	(5.224)
Fraction of observed deaths	0.920	0.922
	(0.272)	(0.269)
Due to infectious/respiratory diseases	0.091	0.091
	(0.287)	(0.288)
Due to cardiovascular diseases	0.569	0.568
	(0.495)	(0.495)
Due to diabetes	0.018	0.018
	(0.132)	(0.134)
Due to cancer	0.156	0.155
	(0.363)	(0.362)
Due to degenerative diseases	0.133	0.135
	(0.340)	(0.342)
Due to other causes	0.034	0.033
	(0.180)	(0.179)
Men	0.451	0.448
	(0.498)	(0.497)
Number of individuals	39,604	69,939
Number of deaths	36,429	64,451
Time at risk	655,383	1,153,862
Ln(Income) Sample, Ages 78–95		
*Post*_*b*_ × *New Health District*_*p*_	0.538	0.253
	(0.499)	(0.434)
Log income, mean real	7.707	7.704
	(0.551)	(0.536)
Men	0.448	0.445
	(0.497)	(0.497)
Number of individuals	38,618	68,224

*Notes*: The sample for *Ln(Income)* includes individuals with zero mean incomes (49 individuals); their incomes are recorded to 1 to perform the analysis. The indicator *Post*_*b*_*× New Health District*_*p*_ denotes treated parishes and cohorts, for which the new local health district established in parish of birth *p* in a year of birth *b* and remained in place in the post-treatment period.

*Source:* Data are from the SIP.

## Results

The analysis starts with the investigation of the contemporaneous effects of the reform. Because I have the data for the number of infants at the parish level for each cohort (from censuses) and the number of individuals who survived to age 78 (from the SIP), I estimate the impact of the reform on the absolute and relative cohort sizes (see Table 3). The results indicate that the reform significantly increased the population of infants. Because this holds for the models controlling for the parish and cohort differences in population size and fertility and because there are no effects of the reform on fertility (as described later), I interpret the results as pointing to the reform-driven benefits for infant survival. Additionally, the results show a significant and sizable increase in the fraction of survivors to age 78 relative to infant population, at 5.0 % to 7.0 % of the mean. Together with the improved environmental conditions, this could indicate that the marginal survivor of the reform to age 78 is negatively selected and that the long-term treatment effects reported later might be underestimated. Although the absence of the high-quality individual data for the whole of Sweden precludes a more detailed analysis, I refer to the study of the local rural area in southern Sweden that explored the effects of health district reform in 1893–1925 on child and infectious disease mortality at the individual level (Lazuka et al. 2016). In line with the results presented here, Lazuka et al. (2016) found that the establishment of health districts led to a more than 50 % decrease in infant mortality and especially in infectious diseases, such as scarlet fever, measles, pneumonia, and diarrhea. These results, as suggested, should be valid for other rural parts of the country.

**Table 3:** Short-term effects of the reform on the cohort size, cohorts 1890–1917, Sweden: Linear fixed-effects regression estimates

	*I*				*M*
	1	2	3	4	5
Ln(Infant Population)					
*Post*_*b*_ × *New Health District*_*p*_	0.0069*	0.0060^†^	0.0076*	0.0081*	0.0044^†^
	(.022)	(.058)	(.011)	(.011)	(.071)
Survivors Aged 78/Infant Population					
*Post*_*b*_ × *New Health District*_*p*_	0.0268*	0.0209^†^	0.0271*	0.0239^†^	0.0177
	(.033)	(.094)	(.031)	(.091)	(.861)
Year of Birth Fixed Effects	Yes	Yes	Yes	Yes	Yes
Parish of Birth Fixed Effects	Yes	Yes	Yes	Yes	Yes
County of Birth × Year of Birth Linear Trends		Yes			
Parish of Birth *X*s × Year of Birth Linear Trends			Yes		
Parish of Birth × Year of Birth Linear Trends				Yes	
Number of Observations (parish of birth × year of birth)	3,278	3,278	3,278	3,278	5,472

*Notes*: *I* = sample of implemented parishes of birth; *M* = sample of matched parishes of birth. The analyses are restricted to parish cohorts used in the sample. The indicator *Post*_*b*_*× New Health District*_*p*_ denotes treated parishes and cohorts, for which the new local health district established in parish of birth *p* in a year of birth *b* and remained in place in the post-treatment period. Share of survivors aged 78 (*Survivors Aged 78/Infant Population*) is calculated as a number of survivors aged 78 relative to infant population size. Data on number of infants (considered to be those below age 2, in order to avoid differences in the timing in enumeration across parishes) are obtained from censuses 1880–1910 at a parish-of-birth × cohort level (census 1890 data are applied to cohorts 1890–1894; census 1900 data, to cohorts 1895–1904; and census 1910 data, to cohorts 1905–1917), and data on the number of individuals survived to the age 78 are from the SIP. Mean of the *Share of Survivors Aged 78* for the untreated is 0.388 (SD = 0.249) for *I* and 0.351 (SD = 0.281) for *M*. *Parish of Birth Xs* denote parish-level pre-treatment control variables and include levels in 1880 and 1890 – 1880 differences for the following variables: log of total population, share of elite and industrial workers in population of men aged 15–55, share of agricultural workers in population of men aged 15–55, mean age of woman, share of women in total population, share of population in labor force aged 15–55, share of married among population aged 15–55, share of infants in total population, share of individuals older than 55 in total population, mortality rate under age 15, share of disabled in total population, mean family size, whether a parish had a railway, and whether a parish had water installations. Model 4 controls for *Parish of Birth* × *Year of Birth Linear Trends*, unlike in models for long-term outcomes with *Parish of Birth* × *Year of Birth Fixed Effects*, because of the limited number of observations per parameter to be estimated. Standard errors (not shown) are clustered at the parish-of-birth level (414 parishes for *I* and 660 parishes for *M*). Numbers in parentheses are *p* values.

^†^*p* < .10; *p < .05

The next analysis turns to long-term outcomes. Table 4 presents results for the long-term effects of the establishment of primary care facilities in the year of birth on mortality from the stratified Cox partial likelihood models. The results show that the treated individuals born within five years after the establishment of the health district are significantly less likely to die between ages 78 and 95, compared with those who were older than age 1 at the reform implementation and born in the same parish. Particularly, they exhibited a 6 % reduction in mortality risk. The estimates are similar in size and robustness for different specifications, such as those including the year-of-birth linear trends across counties of birth, parishes of birth, and pre-treatment characteristics varying across cohorts. In the matched sample, which compares those treated with those untreated in the year of birth, the size of the coefficient indicates a 4.1 % decrease in mortality risk due to the reform. Similar results can be obtained from the Gompertz model and linear probability models, both in terms of the incidence of death and the duration until death (online appendix H). Regarding the overall duration, due to the reform, individuals experienced 4.8 % to 7.7 % longer life until death, which in absolute terms is equivalent to 0.5–0.7 more years alive.

**Table 4:** Long-term effects of the reform on mortality for ages 78–95, cohorts 1890–1917, Sweden: Exponentiated estimates from stratified Cox partial likelihood models (hazard ratios)

	*I*				*M*
	1	2	3	4	5
*Post*_*b*_ × *New Health District*_*p*_	0.940**	0.941**	0.940*	0.948*	0.959*
	(.006)	(.007)	(.035)	(.022)	(.017)
Year of Birth Fixed Effects	Yes	Yes	Yes	Yes	Yes
Parish of Birth Fixed Effects	Yes	Yes	Yes	Yes	Yes
County of Birth × Year of Birth Linear Trends		Yes			
Parish of Birth *X*s × Year of Birth Fixed Effects			Yes		
Parish of Birth × Year of Birth Linear Trends				Yes	
Number of Individuals	39,604	39,604	39,604	39,604	69,939
Number of Deaths	36,429	36,429	36,429	36,429	64,451

*Notes*: Models are adjusted for the left-truncation at age 78. *I* = sample of implemented parishes of birth; *M* = sample of matched parishes of birth. The indicator *Post*_*b*_*× New Health District*_*p*_ denotes treated parishes and cohorts, for which the new local health district established in parish of birth *p* in a year of birth *b* and remained in place in the post-treatment period. *Parish of Birth Xs* denote parish-level pre-treatment control variables and include levels in 1880 and 1890 – 1880 differences for the following variables: log of total population, share of elite and industrial workers in population of men aged 15–55, share of agricultural workers in population of men aged 15–55, mean age of woman, share of women in total population, share of population in labor force aged 15–55, share of married among population aged 15–55, share of infants in total population, share of individuals older than 55 in total population, mortality rate under age 15, share of disabled in total population, mean family size, whether a parish had a railway, and whether a parish had water installations. Standard errors (not shown) are clustered at the parish-of-birth level (414 parishes for *I* and 660 parishes for *M*). Numbers in parentheses are *p* values.

*p < .05; **p < .01

To investigate which causes of death the reform prevented, I explore the estimates for cause-specific mortality in old age in Table 5. The long-term effects on mortality risk from the early-life health care reform are found predominantly in deaths from cardiovascular diseases—mainly acute myocardial infarction and chronic ischemic heart diseases, which contributed most overwhelmingly to the general mortality, at 57 % of all deaths. Dependent on the specification, the individuals treated by the reform had a 4.6 % to 6.1 % lower chance of dying from cardiovascular diseases. The decrease in infectious disease mortality due to the reform is also substantial, between 3.0 % and 10.0 %, albeit not statistically significant. The results suggest that the reform had a substantial impact on cancer mortality risk, decreasing it by approximately 7.8 % to 9.8 %, although this decrease is statistically significant only in the matched sample. Distinguished by sex, the results indicate a significant and strong reduction in infectious/respiratory disease mortality due to the reform and stronger reductions in cardiovascular disease mortality among men compared with those for women (see online appendix I). The estimates in other diagnosis groups are not statistically significant.

**Table 5:** Long-term effects of the reform on cause-specific mortality in ages 78–95, cohorts 1890–1917, Sweden: Exponentiated estimates from stratified Cox partial likelihood models (hazard ratios)

	Infectious/Respiratory	Cardiovascular	Diabetes	Cancer	Degenerative	Other
*I*						
*Post*_*b*_ × *New Health District*_*p*_	0.899	0.939*	0.946	0.922	1.003	0.930
	(.151)	(.035)	(.693)	(.105)	(.963)	(.589)
Year of birth fixed effects	Yes	Yes	Yes	Yes	Yes	Yes
Parish of birth fixed effects	Yes	Yes	Yes	Yes	Yes	Yes
Number of individuals	39,604	39,604	39,604	39,604	39,604	39,604
Number of deaths	3,304	20,733	649	5,675	4,847	1,221
*M*						
*Post*_*b*_ × *New Health District*_*p*_	0.952	0.954*	0.985	0.902*	1.028	1.082
	(.358)	(.044)	(.889)	(.012)	(.553)	(.421)
Year of birth fixed effects	Yes	Yes	Yes	Yes	Yes	Yes
Parish of birth fixed effects	Yes	Yes	Yes	Yes	Yes	Yes
Number of individuals	69,939	69,939	69,939	69,939	69,939	69,939
Number of deaths	5,862	36,579	1,185	9,961	8,723	2,141

*Notes*: Models are adjusted for the left truncation at age 78. *I* = sample of implemented parishes of birth; *M* = sample of matched parishes of birth. The indicator *Post*_*b*_*× New Health District*_*p*_ denotes treated parishes and cohorts, for which the new local health district established in parish of birth *p* in a year of birth *b* and remained in place in the post-treatment period. Standard errors (not shown) are clustered at the parish-of-birth level (414 parishes for *I* and 660 parishes for *M*). See the main text for further description. Numbers in parentheses are *p* values.

*p < .05

The estimates for natural logarithm of income, presented in Table 6, suggest the positive effects of the reform on long-term incomes. With the full follow-up period between ages 78 and 95, the treated individuals have a 2.0 % higher income compared with their counterparts. The estimates are stable within a range of 1.6 % and 2.5 % across different specifications, including those with parish-specific year-of-birth trends or exact matching. Because individuals progressively died from the beginning of the observation with age, I check the results for shorter follow-ups and a mean residual income. These results show estimates of a similar or slightly larger magnitude (see online appendix J). Additionally, I exclude person-years under widowhood from the calculation of the mean income, and the estimates increase in magnitude up to 3.0 %. The results also remain essentially unchanged when I exclude first early cohorts, who were covered by a different pension system. I refrain from presenting the income effects in absolute terms because the earnings across cohorts under analysis vary considerably.

**Table 6:** Long-term effects of the reform on natural logarithm of income for ages 78–95, cohorts 1890–1917, Sweden: Linear fixed-effects regression estimates

	*I*				*M*
	1	2	3	4	5
*Post*_*b*_ × *New Health District*_*p*_	0.0203*	0.0212*	0.0228*	0.0246*	0.0160*
	(.041)	(.042)	(.028)	(.033)	(.035)
Year of Birth Fixed Effects	Yes	Yes	Yes	Yes	Yes
Parish of Birth Fixed Effects	Yes	Yes	Yes	Yes	Yes
County of Birth × Year of Birth Linear Trends		Yes			
Parish of Birth *X*s × Year of Birth Fixed Effects			Yes		
Parish of Birth × Year of Birth Linear Trends				Yes	
Number of Individuals	38,618	38,618	38,618	38,618	68,224

*Notes*: *I* = the sample of implemented parishes of birth; *M* = sample of matched parishes of birth. The indicator *Post*_*b*_*× New Health District*_*p*_ denotes treated parishes and cohorts, for which the new local health district established in parish of birth *p* in a year of birth *b* and remained in place in the post-treatment period. *Parish of Birth Xs* denote parish-level pre-treatment control variables and include levels in 1880 and differences 1890–1880 for the following variables: log of total population, share of elite and industrial workers in population of men aged 15–55, share of agricultural workers in population of men aged 15–55, mean age of woman, share of women in total population, share of population in labor force aged 15–55, share of married among population aged 15–55, share of infants in total population, share individuals older than 55 in total population, mortality rate under age 15, share of disabled in total population, mean family size, whether a parish had a railway, and whether a parish had water installations. Standard errors (not shown) are clustered at the parish-of-birth level (414 parishes for *I* and 660 parishes for *M*). Numbers in parentheses are *p* values.

*p < .05

**Fig. 2:**
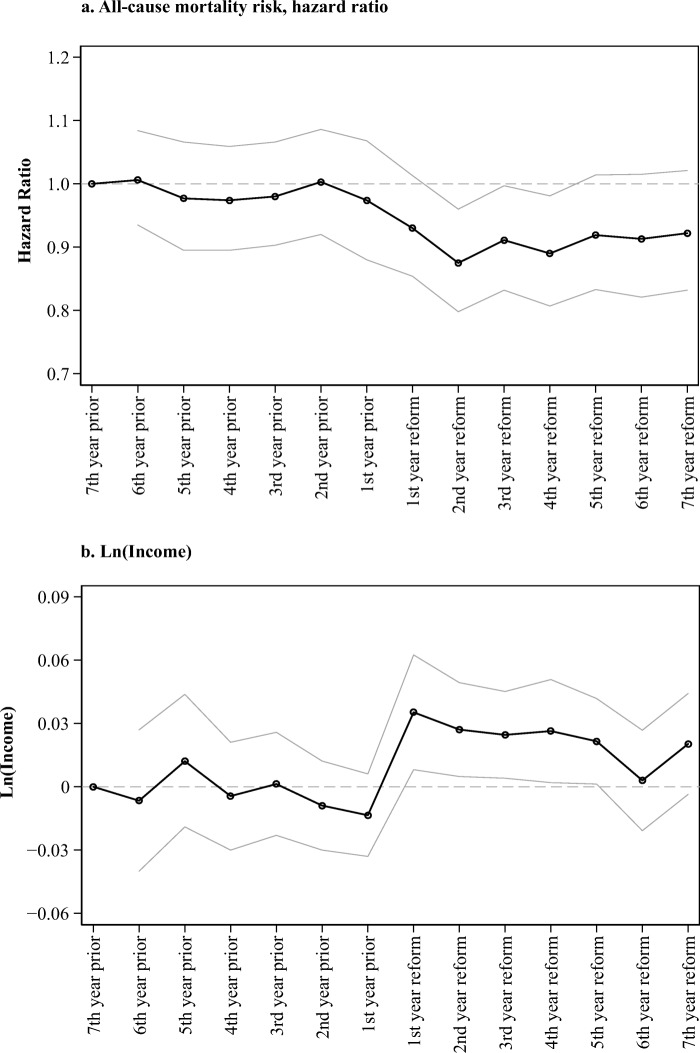
Coefficient estimates for the long-term effect before and after (seven years with a new health district in place) the reform on mortality (panel a) and natural log of income (panel b). The coefficients are estimated for those born in the *implemented* parishes. Those born seven years prior to the reform are the reference category. The figure presents exponentiated coefficients from stratified Cox partial likelihood models for mortality and linear fixed-effects regression estimates for natural log of income. Standard errors (not shown) are clustered at the parish-of-birth level (418 parishes). Point estimates and 95 % confidence intervals are shown.

I further investigate the long-term effects with an event study design (Fig. 2). Using data for the implemented sample, the figure plots the coefficient estimates obtained from replacing *Post*_*b*_ with a series of year-of-birth dummy variables and omitting individuals born seven years prior to the establishment of the health district (they were age 7 at that time). As expected, I observe a sharp change in the coefficients beginning from the first year of the reform implementation and afterward, pointing to the benefits for those who were in infancy at the time of the reform implementation. Clearly, there are no policy effects for older child ages (ages 1–7). A slightly pronounced effect for individuals age 1 is likely to emerge because the dates of establishment of districts are defined as years, so individuals who are born in the second half of the year were still in their infancy at the start of the district. Noteworthy, the effects are especially strong one year after the opening of a district, consistent with the employment of midwives who were usually recruited after the doctor began to serve the district. The effects become smaller after age 5, pointing to the additional benefits of the reform among pupils, related to the coincidental increase in the investment into primary schooling or doctor-enforced school closures during epidemic outbreaks. The results indicate no diverging health and income trajectories in the pre-reform years. The pattern looks similar for the matched sample, for which the effects emerge only from the first year of the reform implementation.

These estimates represent the intention-to-treat estimates, and I do not directly observe the probability that the individual received the reform treatment. In the models, all individuals born in the parishes where a new health district was opened are considered as treated, and the effects are therefore larger if any smaller proportion of them was treated. A similar set of public health initiatives has been studied for a small local area in southern Sweden that provided a detailed record of participation rates (Lazuka 2018b; Lazuka et al. 2016). Regarding isolation and disinfection measures undertaken by the provincial doctors, the initial disease panorama affected the take-up probability. Roughly one-half of infants born in new health districts reacted to the isolation initiatives, mainly because of decreases in mortality from airborne infectious diseases. In 1890–1920, no more than one-half of all midwives working throughout the country had a license; in the countryside, the proportion of births attended by these midwives did not exceed one-fifth (Statistiska Centralbyrån 1880–1910, 1911–1920). Therefore, to transform the intention-to-treat estimates of mortality and income into the treatment effects on the treated, the former should be divided by a proportion in the range of 0.2 to 0.5.

Table 7 distinguishes long-term effects by subgroups based on the reform components and socioeconomic characteristics of the parishes. The estimates for the heterogeneous effects, from models in which the implementing parishes are divided into two groups by the public health investment and additional midwives and doctors employed per parish population, support the notion that improving the intensity of the reform produces more benefits. When parishes are distinguished by the share of infants and mortality rates under age 15, the health effects are observed for all parishes but are larger at higher baseline levels of disease. Consistent with the observation that men were more sensitive to infectious disease in infancy (e.g., infant mortality was 4 % higher among men across study cohorts), point estimates for both all-cause mortality and natural logarithm of income are larger for them than for women. Regarding the role of treatment in inequality development, the estimates for both outcomes suggest that individuals from all socioeconomic backgrounds benefited from the reform, but the benefits were greater for the more disadvantaged groups. The reform led to at least 2.4 % and 10.0 % decreases in all-cause mortality risks and 0.5 % and 3.0 % increases in incomes for individuals with wealthier and poorer socioeconomic origins, accordingly.

**Table 7:** Long-term effects of the reform on mortality and natural logarithm of income by subgroups, ages 78–95, cohorts 1890–1917, Sweden: Exponentiated estimates from stratified Cox partial likelihood models for mortality risk (hazard ratios), adjusted for left-truncation at age 78, and linear fixed-effects regression estimates for ln(income)

	Mortality Risk	Ln(Income)	Mortality Risk	Ln(Income)
	Investments Into Health Care per Capita		Mortality Rate Under Age 15	
*Post*_*b*_ × *New Health District*_*p*_ × *Low*_*p*_	0.949*	0.0275*	0.963	0.0107
	(.048)	(.034)	(.216)	(.411)
*Post*_*b*_ × *New Health District*_*p*_ × *High*_*p*_	0.935**	0.0158	0.923*	0.0097
	(.004)	(.109)	(.015)	(.431)
	Doctors Employed per Capita		SES, Share of Skilled	
*Post*_*b*_ × *New Health District*_*p*_ × *Low*_*p*_	0.943*	0.0258^†^	0.900**	0.0297
	(.018)	(.041)	(.005)	(.135)
*Post*_*b*_ × *New Health District*_*p*_ × *High*_*p*_	0.940**	0.0185*	0.972	0.016
	(.009)	(.066)	(.331)	(.103)
	Midwives Employed per Capita		SES, Share in Labor Force	
*Post*_*b*_ × *New Health District*_*p*_ × *Low*_*p*_	0.951*	0.0167	0.896**	0.0431*
	(.045)	(.163)	(.002)	(.029)
*Post*_*b*_ × *New Health District*_*p*_ × *High*_*p*_	0.931**	0.0234*	0.976	0.0049
	(.003)	(.024)	(.400)	(.637)
	Share of Infants		Mean Family Size	
*Post*_*b*_ × *New Health District*_*p*_ × *Large*_*p*_	0.896**	0.0195	0.932*	0.0187
	(.001)	(.225)	(.027)	(.200)
*Post*_*b*_ × *New Health District*_*p*_ × *Small*_*p*_	0.973	0.0247^†^	0.941*	0.0180
	(.353)	(.063)	(.049)	(.179)
	Men		Railway	
*Post*_*b*_ × *New Health District*_*p*_ × *Yes*_*p*_	0.929**	0.0249	0.970	0.0112
	(.003)	(.183)	(.250)	(.297)
*Post*_*b*_ × *New Health District*_*p*_ × *Otherwise*_*p*_	0.950*	0.0164	0.886**	0.0371*
	(.043)	(.136)	(.004)	(.049)
Year of Birth Fixed Effects	Yes	Yes	Yes	Yes
Parish of Birth Fixed Effects	Yes	Yes	Yes	Yes
Number of Individuals	39,604	38,618	39,604	38,618
Number of Deaths	36,429		36,429	

*Notes*: The indicator *Post*_*b*_*× New Health District*_*p*_ denotes treated parishes and cohorts, for which the new local health district established in parish of birth *p* in a year of birth *b* and remained in place in the post-treatment period. *Investments Into Health per Capita, Doctors and Midwives Employed per Capita* are at the level of health district. Other variables are at the level of parish of birth; census 1890 data are applied to cohorts 1890–1894; census 1900 data are applied to cohorts 1895–1904, and census 1910 data are applied to cohorts 1905–1917. To investigate heterogeneous treatment effects, triple differences are introduced (additional terms into Eq. (1), such as *Post*_*b*_*× New Health District*_*p*_*× Subgroups*_*p*_, which is an indicator for the health district established in parish *p* in the year of birth *b* and subgroup of parishes *s*, and the underlying two-way interactions and main effects). All groups, except for *Men* and *Railway*, are divided at the median. For *Men*, results are for men (yes) and women (otherwise). Standard errors (not shown) are clustered at the parish-of-birth level (414 parishes). Numbers in parentheses are *p* values.

†*p* < .10; *p < .05; **p < .01

## Robustness Analyses

Given the rise in overall social spending starting from the mid-1880s, one might question whether the effects captured are entirely due to the health care reform and not due to other overlapping societal initiatives. As mentioned earlier, the local governments put in place no other public health measures, such as improvements in water facilities, vaccinations, or food hygiene control, until the 1930s. From the data set, I observe that increases in public health investment associated with the introduction of health districts also implied increases in overall social spending. A more careful look suggests that the overwhelming part of the residual local investment was spent on primary schools, which do not directly affect infants or health for those under age 5. Additional resources were also accumulated for poor relief, although they covered only 1.2 % of children under age 15 with direct support (Statistiska Centralbyrån 1880–1917). Nevertheless, I reestimate the models by adding pre-treatment trends for health districts based on investments in primary schooling, infrastructure, and poor relief (interacted with year-of-birth dummy variables). It is evident (Table 8, panel A) that the rise in social expenditures other than public health care is not responsible for the results.

**Table 8:** Robustness analyses for the long-term effects of the reform on mortality and natural logarithm of income at ages 78–95, cohorts 1890–1917, Sweden: Exponentiated coefficients from stratified Cox partial likelihood models for mortality (except for panel D), adjusted for left-truncation at age 78, and linear fixed-effects regression estimates for ln(income)

	Mortality Risk	Ln(Income)	Mortality Risk	Ln(Income)
A. Adding Investment Into Education, Infrastructure, and Poor Relief			B. Adding Pretreatment Number of Residents With Voting Rights per Capita	
*Post*_*b*_ × *New Health District*_*p*_	0.932**	0.0286**	0.940**	0.0187^†^
	(.002)	(.006)	(.004)	(.061)
Number of individuals	39,604	38,618	39,604	38,618
Number of deaths	36,429		36,429	
Parishes of birth	414	414	414	414
C. Correcting for Survival Bias Using Heckman Two-Stage Procedure			D. Correcting for Survival Bias Following van den Berg and Drepper	
*Post*_*b*_ × *New Health District*_*p*_	0.940**	0.0200*	0.935**	––
	(.006)	(.044)	(.002)	––
Number of individuals	39,604	38,618	39,604	
Number of deaths	36,429		36,429	
Parishes of birth	414	414	414	
E. Excluding Counties of Birth Affected by Emigration to the United States			F. Adding Controls for Coinciding Unfavorable Health and Economic Conditions	
*Post*_*b*_ × *New Health District*_*p*_	0.938**	0.0186^†^	0.937**	0.0220*
	(.006)	(.081)	(.007)	(.027)
Number of individuals	36,023	35,134	39,604	38,618
Number of deaths	33,127		36,429	
Parishes of birth	385	385	414	414
G. Excluding Counties of Birth Affected by WWI			H. Adding Parish-Specific Controls for Age Structure	
*Post*_*b*_ × *New Health District*_*p*_	0.933**	0.0248*	0.943**	0.0208*
	(.004)	(.018)	(.009)	(.024)
Number of individuals	35,320	34,453	39,604	38,618
Number of deaths	32,391		36,429	
Parishes of birth	409	409	414	414
I. Adding Parish-Specific Controls for Local Labor Market and Migrant Structure			J. Excluding Counties of Residence Affected by Spanish Flu	
*Post*_*b*_ × *New Health District*_*p*_	0.930**	0.0251*	0.938*	0.0225*
	(.004)	(.016)	(.010)	(.043)
Number of individuals	39,604	38,618	32,212	31,433
Number of deaths	36,429		29,482	
Parishes of birth	414	414	411	411
Year of Birth Fixed Effects	Yes	Yes	Yes	Yes
Parish of Birth Fixed Effects	Yes	Yes	Yes	Yes

*Notes*: Panel D shows exponentiated shared gamma-frailty (Weibull baseline distribution) model estimates, adjusted for left truncation at age 78. The indicator *Post*_*b*_*× New Health District*_*p*_ denotes treated parishes and cohorts, for which the new local health district established in parish of birth *p* in a year of birth *b* and remained in place in the post-treatment period. In panel E, Värmland and Halland counties are excluded as the primary emigrant counties, where the countryside experienced mass migration to the United States. In panel G, Norrbotten and Västerbotten counties are excluded because most were affected by World War I. In panel J, Koppaberg, Gävleborg, Västernorrland, Jämtland, Västerbotten, and Norrbotten counties are excluded because most were affected by the Spanish flu. For further details, see online appendix K. Standard errors (not shown) are clustered at the parish-of-birth level. Numbers in parentheses are *p* values.

^†^*p* < .10; *p < .05; **p < .01

So far, long-term estimates appear to have been robust to controlling for observable and unobservable pre-treatment factors specific to the location of birth. Online appendix K contains a detailed description of the additional robustness checks. First, the main results are qualitatively similar when I consider larger pre- and post-treatment periods, such as 7 or 10 years. Next, I try to include more factors measuring local leadership, possibly explaining the differences between the localities with different timing of the reform implementation (Table 8, panel B). Also, I explicitly account for selective survival in the models by applying a two-stage Heckman (1979) correction procedure (Table 8, panel C) and van den Berg and Drepper (2011) approach proposing to estimate shared-frailty models with left-truncated data (Table 8, panel D). An additional concern is whether any discontinuous changes in specific locations coincided with the timing of the health care reform, such as emigration to the United States (panel E) or unfavorable health and economic conditions in general (panel F), events related to the World War I (panel G), internal migration (panel H), any other events related to particular age groups (panel I), and Spanish flu (panel J), thereby changing the composition of the individuals’ parents or of the individuals toward wealthier or healthier ones. I perform a bounds exercise as an additional account of the potential bias due to selective emigration to the United States, which primarily targeted rural areas that could be the reform adopters, based on data on the shares of the poor emigrants to the United States (Statistiska Centralbyrån 1890–1910). This exercise, presented in detail in online appendix K, shows that the estimated effects are fairly stable and statistically significant in all subsamples. Finally, it is important to test whether the reform by itself affected the composition of the parents and fertility. The analyses successfully pass all aforementioned robustness checks.

## Discussion and Conclusions

To date, knowledge of the long-term influence of the public health reforms initiated across developed countries during several decades surrounding the turn of the twentieth century has been extremely scarce. This period was characterized by huge social investments in reforms applying the newly arrived bacteriological knowledge and reductions in infectious disease burden, with plausible consequences for the subsequent cohorts. Using a DiD approach, I examine the long-term effects of the universal expansion of health districts in rural areas of Sweden between 1890 and 1917 in the year of birth of the individuals on their pension and capital income and health at ages 78–95. I find effects for oldest-old mortality and income, and these effects are robust to a variety of alternative hypotheses. The health district reform was designed to provide all parts of Sweden with access to primary care, giving this opportunity equally to the disadvantaged regions, and became one of the first elements of the modern welfare state. Due to the reform, individuals treated in the year of birth attained approximately 4.2 % to 6.0 % decreases in all-cause mortality risk, roughly equivalent to 0.5–0.7 additional years spent alive and to 31 % to 43 % of overall cohort improvements. The positive effects on the total income amount to 1.6 % to 2.5 %. These reduced-form effects are sizable and appear to be similar to those demonstrated in previous studies relying on either positive or negative early-life shocks and focusing on the elderly (e.g., Aizer et al. 2016; Myrskylä et al. 2013; see comparison in online appendix L).

Previous literature has pointed to infectious diseases, nutritional deficits, childhood poverty, and stressful family conditions in early life as those strongly related to longer-term outcomes (Lynch and Davey Smith 2005). In proposing that cohorts born around the time of large social investments in 1870–1930 should have strong benefits in elderly ages, Fogel (1994) listed their various types of investments rather than pointing to the decisive one. The current study suggests the link between prevention of infectious disease due to the Swedish primary care reform and old-age survival and income benefits. First, I find that the reform is likely to have improved infant survival contemporaneously. Second, in the long term, the event study analysis shows no effect of the reform beyond the year of birth, in which susceptibility to infectious disease is the largest, thus pointing to it as to the critical period. Third, the results show that the reform mainly reduced oldest-age mortality from cardiovascular disease, in line with interpretations by Finch and Crimmins (2004) and the chronic inflammation hypothesis. This study further demonstrates that health benefits translate into acquired incomes and that this effect is likely to occur directly, given that the rural cohorts under study did not differ in the acquired mandatory primary schooling (Ekstedt 1976). Finally, the analysis of the heterogeneous effects supports the link to both disease burden and medical personnel imposing control of infectious disease.

The study results indicate that implementation of the health care reform, through improved infant health, led to larger effects for individuals with poorer socioeconomic backgrounds captured as far as in oldest-old ages. In line with the expectations, given the design of the health care reform, I find that the local preventive initiatives generated long-term benefits across all subpopulations. This study provides several indications in support of the notion that health policy helped to reduce intergenerational disparities in health and incomes (Case et al. 2002; Currie and Schwandt 2016). Primarily, in relative terms, individuals from lower socioeconomic classes enjoy larger effects in both longevity and long-term incomes. Similarly, in absolute terms, more generous public health investments per capita devoted to the creation and functioning of the health district generated stronger individual responses. Such differences emerge in the treatment effects because the utilization of health services occurred equally across families with different socioeconomic characteristics.

In the most conservative terms, the public costs of the reform studied here, including expenditures on health care maintenance in parishes not affected by the reforms, constitute a tiny share of the national income between 1890 and 1917 (author’s own calculations). To calculate the social rate of return to the reform, I compare the difference in the discounted incomes at ages 78–95, adjusted with improvements in both survival probabilities and incomes, with the public health investments. I obtain 1890–1917 cohort survival probabilities for ages 78–95 for Sweden from the Human Mortality Database (2017), cohort- and age-specific incomes at ages 78–95 from the SIP, and the real interest rate (2.6 %) from Waldenström (2014). The discount rate is based on the interest rate compounded by each of the years 78–95. With such a distance between the timing of investments and the ages at which the reduced-form effects are captured, a social rate of return equals a ratio of 6 to 1. In addition to documenting distinct economic returns, this article points to the causation between public health investments in the year of birth and later reductions in chronic diseases and the necessity for their public provision in order to restrain the future costs related to the support of the aging population, providing the relief that could last for the entire lifespan.

## Electronic Supplementary Material

ESM 1 (PDF 387 kb)
