# Supplementary material for: Early-Life Assets in Oldest-Old Age: Evidence From Primary Care Reform in Early Twentieth Century Sweden
Source: Demography. 2019 Jan 16;56(2):679–706. doi: 10.1007/s13524-018-0758-4 (PMC6449494; doi:10.1007/s13524-018-0758-4)
Supplement: Supplementary file 1 — (PDF 387 kb) [file 13524_2018_758_MOESM1_ESM.pdf]

ONLINE APPENDICES

to the paper by Lazuka, Volha

**“Early Life Assets in Oldest-Old Age:
Evidence From Primary Care Reform in Early Twentieth Century Sweden”**

Appendix A

Context of the 1890 Health District Reform

In Sweden, economic development had accelerated by the last quarter of the nineteenth century. According to recent estimates, in per capita terms, the real GDP grew at an annual rate of 1% prior to 1890, and throughout the twentieth century this rate was constant at 2% (Schön and Krantz 2012). The last subsistence crisis occurred in Sweden in the late 1860s (Jörberg 1994). Beginning from 1880, employment in industry increased from 15 to 35%, although by the 1920s, approximately one half of the population still worked in agriculture (Statistiska Centralbyrån 1969). In the following decades, the Swedish manufacturing sector became dominant and services expanded. The same development is observed in the real wages of workers, which began to gradually increase in 1880, and accelerated towards the middle of the twentieth century (Jörberg 1972). Distinctly for Sweden, the majority of industrial workers were employed and lived in rural industrial locations. Urbanisation followed a similar pattern. The urbanisation rate was slow prior to 1890 and afterwards amounted to 2% per year (Statistiska Centralbyrån 1999). Despite this increase, by the first half of the twentieth century, the majority of the Swedish population was still rural. According to international standards, Swedish cities were small, with the population of the biggest cities, such as Stockholm and Gothenburg, no more than 500, 000, which accounted for less than one-tenth of the total population. The share of population that resided in the countryside (*land*), and therefore outside cities (*stad*) or semi-urban locations (*köping*), declined only from 80 to 70% between 1890 and 1920 and to less than one-half of the total afterwards (Statistiska Centralbyrån 1999).

The improvements in population health in Sweden exhibited a similar pattern. There was no trend in change in life expectancy at birth until the middle of the nineteenth century, whereas afterwards it increased almost linearly from age 42 to 78 (Bengtsson 2006). Human stature also increased, although according to some estimates these changes were delayed until 1880 (Sandberg and Steckel 1997). The Swedish population increased rapidly, from approximately 4.2 to 5.9 million between 1880 and 1920 and doubled by the turn of the century (Statistiska Centralbyrån 1999). Prior to the first quarter of the twentieth century, such rapid population growth was largely a result of declining death rates, mainly among children and infants, from airborne and foodborne infectious diseases such as whooping cough, measles, diphtheria and diarrhoea (Preston et al. 1972). Between 1880 and 1920, the infant mortality rate declined dramatically, from 129 to 72 per 1000, and death rates in the 1–15-year-old population fell even more rapidly, from 13 to 4 per 1000 children (Statistiska Centralbyrån 1999). Among the primary demographic processes contributing to the slowdown in population growth in this period, several stand out. These include the Spanish flu, which killed slightly less than one per cent of the population and scarred many more, gradually falling birth rates, and mass emigration to the United States, which subtracted approximately a million residents (van Hofsten and Lundström 1976). In later decades, the improvements in life expectancy have been attributed to the decrease in death rates among the working population and the elderly. Regarding the causes of death, in this period the most dramatic reductions were witnessed in mortality rates from pneumonia, degenerative diseases of organs and tissues, and vascular and heart diseases (Preston et al. 1972; Statistiska Centralbyrån 2010).

The Swedish authorities recognised the need for the provision of public health in the nineteenth century. After the establishment of the national vital statistics in 1749, the medical board produced several reports about mortality in the country and underlined the necessity to prevent deaths from smallpox and other infectious diseases (Johannisson 2006). Under the need to save the labour force, at the beginning of the nineteenth century, the government set up the foundation for public health care, which introduced compulsory vaccination against smallpox and obliged the parishes to open

poorhouses. Local hospitals and physicians focussed much less on these measures, although their free public offer became assured. However, with regard to the overall panorama of infectious diseases, any public measures before the 1880s remained responsive. For many years, under the fear of epidemics spreading to neighbouring countries, the authorities imposed quarantine regulations and the inspection of cargo, in addition to mobilising medical practitioners (Bourdelais 2002). In the mid-nineteenth century, such initiatives covered only the major Swedish towns, which by that time were equipped with hospitals and medical personnel. However, the epidemics appeared to fail any broad and costly quarantine and surveillance efforts in the urban localities and killed many more in rural areas, where no measures were in place (Niemi 2007). By disentangling the mode of transmission of infectious diseases and the causal agents, the international bacteriological discoveries in the 1870–1880s helped to target public efforts and provided tools to combat disease. Consequently, the governmental authorities had to admit the necessity for a radical programme of public health care provision throughout the entire country.

From 1890 until the 1920s, all communities in Sweden gradually received access to public health care in the form of local health districts. The institute of a provincial doctor district (*provinsialläkardistrikt*), which is organised around an assigned doctor, midwives and a hospital, dates back to 1773. Until the mid-nineteenth century, the number of centrally introduced health districts amounted to 2 per 100,000 inhabitants and disproportionally covered the more urbanised locations (Medicinalstyrelsen 1907). In 1840, the industrial elite were granted the right to organise a local medical district serving their residencies. After establishing a community council representative of all taxpayers, a few decades later, the local government reform extended this right to all parishes (Lindblom 1967). The local health administration instructions followed shortly, which prescribed each parish or group of parishes to set up a public committee, including a magistrate and a doctor, to address public health matters, in particular in controlling the spread of infectious diseases. The location-initiated creation of health districts accelerated accordingly, beginning in the 1880s. However, without any government subsidies, between 1840 and the late 1880s, the process of expansion of provincial doctor districts was sluggish and favoured wealthy and industrialised locations (Medicinalstyrelsen 1907). Driven both by international achievements in municipal governments and medicine and by rapid industrial and population growth in the countryside, in 1890 the Swedish state authorities announced a reform aimed at creating medical districts in all parts of the country, giving this opportunity equally to economically disadvantaged areas. According to the reform, each group of parishes with 8,000–12,000 inhabitants applying for a public health district could be subsidised with 1,500 SEK from the government and had to accumulate 2,500 SEK from local sources. Additionally, the state began to stimulate the graduation of young medical professionals and attract them to rural parishes, primarily by guaranteeing career promotions and public pensions. The reform therefore was designed centrally to bring access to public health care, with more local resources devoted to it in the rural populations.

To identify the initiatives undertaken in the parishes due to the 1890 reform, I studied the yearly reports of the provincial district doctors 5 years before and after the reform, available in the National Archives (Medical History Database 1885–1900; Riksarkivet 1885–1914). To do this, I drew 20% random samples of two types of health districts: established in 1890–1917 (25 districts) and – for a baseline comparison – those established prior to 1881 (33 districts) and studied them in the archives or digitally. In general, the yearly reports are coherent in suggesting specific actions in general care after the opening of primary care facilities, all intended to prevent the spread of infectious diseases. Due to the bacteriological discoveries in the 1870–1880s, the intervention was able to control the spread of disease in localities, although no cures or vaccines were available until the late 1930s.

One set of initiatives was related to intensified monitoring, tracing and notification of infectious disease by doctors, including service trips to the villages with disease outbreaks, diagnostics of the

samples in the city laboratories and new doctor or nurse appointments in case of severe epidemics. Another set of initiatives included forced isolation of the sick family members from the rest of the family or the whole family from the rest of the village in epidemic hospitals or rooms, and disinfection of the rooms and belongings with disinfection apparatuses. The cottage hospitals or health stations in the parishes were built for this reason and epidemic nurses were employed, whereas chronic patients for many years were delivered for in-patient care to the neighbouring cities. However, a closer look at the pre-treatment periods and of the comparison health districts suggests that disease notification, isolation and disinfection was in practice in the Swedish countryside since 1893. More specifically, in response to the cholera epidemic, instructions dealt with sick passengers on the railroads by means of disinfecting coaches and isolating affected persons at the nearest hospital (Svensk författningssamling 1892: 67). Additionally, a state law of 1893 required each populous commune to establish a hospital that could be used for the obligatory isolation of sick residents and travellers (Svensk författningssamling 1893: 61). Provincial doctors indicate that it was usually difficult to achieve isolation of a sick family member from the rest of the family, because the family usually occupied one room (in winters, together with livestock). The same holds for the recommendation to avoid overcrowding at burials, in case of death due to the infectious disease. In larger and more populous parishes, where epidemic hospitals and apartments were set up, this could be more easily achieved with forcing measures. Therefore, what the reform brought in regarding disease notification, isolation and disinfection was probably their intensified usage.

A new initiative undertaken during the reform was encouragement of school closures during the epidemic outbreaks, such as scarlet fever, measles, diphtheria, whooping cough and others. Each report indicated such enforcement measures and their implementation. Finally, provincial doctors, by themselves being young graduates from the universities, stimulated the employment of new midwives giving priority to modern medical knowledge over the limited practical experience. Doctors often stressed it more particularly: noted that they encourage employments of midwives ‘during the year graduated’ as opposed to older midwives due to the ‘enormous differences in antiseptics and other medical knowledge taught by old and new schools’ (Riksarkivet 1885–1914). While previously midwives by themselves could be carriers of disease, under the control of medical doctors and accompanied by the introduction of the disinfection instructions for childbirth in 1881, the organisation of health districts encouraged the employment of midwives highly competent in the use of antiseptics, emergency, preventive and supportive care (Lazuka 2018). In addition to the use of antiseptics, these midwives were able to recognise infectious diseases, monitored the health of the mother and a new-born shortly after birth, and encouraged early breastfeeding, treatment of umbilicus and isolation of infants from the rest of the family. Quackery was highly prohibited by the new doctors. Additionally, the medical practitioners brought surveillance and relief from the disease.

From the yearly reports, several spheres of public health emerge where doctors did or could not take any action. Initiatives did not cover water supply and sewerage treatments, and while there are frequent notes about bad water resulting from factory or dairy farm activities, in general, water quality was regarded as satisfactory. Measures to control the quality of food and nutritional habits were also scarce. After 1910, local health inspectors occasionally took steps to improve hygienic conditions in the slaughterhouses, grocery stores, and dairies across the area. The pasteurization of milk was made compulsory around 1940. Doctors indicated that inhabitants treated themselves with coffee and cognac against disease, children fed with coffee in their early diets, but claimed that diets, prevailed in grain and pork, were nutritious and satisfactory. The same holds for housing conditions, which were unsanitary, overcrowded and dirty. In those years, municipal counties considered that no actions needed to be done in regard of removal of slops and sewage, and overcrowding and dirtiness were regarded as common features of rural life. It was only since the 1920s that parishes built public bathhouses and

several decades later housing conditions began to be improved. The reports discuss the availability of the apothecary in the area and note that there are their substitutes in the form of apothecary boxes delivered to the doctors' stations, although agreed on them making little difference due to the absence of drugs against the infectious disease.

While no actions in improving socio-economic conditions of the parishes and families were undertaken due to the reform, there are several indications that primary care initiatives did not correlate with socio-economic characteristics of the parishes nor, supported in the recent study of a local rural area in Sweden (Lazuka et al. 2016), with regard to the socio-economic background of a new-born child. This was expected, as health care was provided to the public through redistribution for no or negligible cost to the recipients (Curtis 2011). Based on the case of the local rural area in Sweden, we know that establishment of health districts in 1890-1925 led to a more than 50% decrease in infant mortality and especially in infectious diseases, such as scarlet fever, measles, pneumonia, and diarrhoea (Lazuka et al. 2016). Isolations and disease notification measures, practiced in each populous rural parish since the 1890s, became efficient in reducing mortality in the ages 1-5. With regard to wealth, the parishes that established the reform earlier were likely to be on average poorer compared to later adopting parishes, indicated, for example, by real total investment or a fraction of the active population in the labour force prior to the reform (see Table 1 of the main text). This feature is probably unique to Swedish primary care policies because water purification technologies, implemented in cities of Sweden or in other developed countries of Europe and North America in the past led to much quicker reform adoption among affluent communities (cf. Drangert et al. 2002; Kesztenbaum and Rosenthal 2017).

References (to the sources not cited in the main text)

Bengtsson, T. (2006). *Perspectives on Mortality Forecasting. 3. The Linear Rise of Life Expectancy: History and Prospects,* edited by T. Bengtsson. Stockholm: Swedish National Social Insurance Board.

Bourdelais, P. (2002). Cholera: A victory for medicine? In R.S. Schofield, D. Reher, & A. Bideau (Eds.), *The Decline of Mortality in Europe* (pp. 118–130). Oxford: Clarendon Press.

Drangert, J., Nelson, M.C., & Nilsson, H. (2002). Why did they become pipe-bound cities? Early water and sewerage alternatives in Swedish cities. *Public Works Management & Policy,* 3, 172–185.

Johannisson, K. (2006). The people's health: Public health policies in Sweden. In D. Porter (Ed.), *The History of Public Health and the Modern State* (pp. 165–182). Amsterdam-Atlanta: GA Rodopi.

Jörberg, L. (1972). *A History of Prices in Sweden, 1732-1914.* Lund: Lund University.

Jörberg, L. (1994). Structural Change and Economic Growth in Sweden in the Nineteenth Century. In R.A. Church, E.A. Wrigley, L. Jörberg, & P.K. O'Brien (Eds.), *The Industrial Revolutions: The Industrial Revolution in Europe* (pp. 411–545). Oxford: Blackwell.

Kesztenbaum, L., & Rosenthal, J. (2017). Sewers' diffusion and the decline of mortality: The case of Paris, 1880–1914. *Journal of Urban Economics*, 98, 174–186.

Niemi, M. (2007). *Public Health and Municipal Policy Making. Britain and Sweden, 1900-1940.* Aldershot: Ashgate.

Preston, S.H., Keyfitz, N., & Schoen, R. (1972). *Causes of Death: Life Tables for National Populations*. New York: Seminar Press.

Sandberg, L., & Steckel, R.H. (1997). Was industralization hazardous to your health? Not in Sweden. In R.H. Steckel & R. Floud (Eds.), *Health and Welfare during Industrialization* (pp. 127–159). Chicago: University of Chicago Press.

Statistiska Centralbyrån. (1969). *Historisk Statistik för Sverige. Del 1. Befolkning 1720–1967 [Historical Statistics for Sweden. Part 1. Population 1720–1967]*. Stockholm: KL Beckmans Tryckerier AB.

Statistiska Centralbyrån. (1999). *Befolkningsutvecklingen under 250 År: Historisk Statistik för Sverige [Population Development for 250 Years: Historical Statistics for Sweden]*. Halmstad: Bulls Tryckeri.

Statistiska Centralbyrån. (2010). *Cohort Mortality in Sweden: Mortality Statistics since 1861*. Stockholm: Statistiska Centralbyrån.

Svensk författningssamling (1892): 67 *[Swedish Code of Statutes 1892: 67*].

van Hofsten, E.A., & Lundström, H. (1976). *Swedish Population History. Main Trends from 1750 to 1970*. Stockholm: Statistiska Centralbyrån.

Appendix B

Description of data sources for health districts and parishes of birth

The data on the division of parishes into health districts and its changes have been gathered from several sources. Primarily, governmental reports on provincial doctor districts contain detailed data on the allocation of the parishes as well as the creation dates and funding of the new districts collected from the health board acts (Medicinalstyrelsen 1907, 1939). I additionally verified these divisions with several sources, such as the provincial doctor reports attained from the National Archive in Sweden (Riksarkivet 1893-1946), statistical yearbooks on health care (Statistiska Centralbyrån 1880-1910; 1911-1920) and on public health investment (Statistiska Centralbyrån 1880-1917). These sources provided information on the number of the medical personnel employed, such as doctors and midwives, and public spending, both in health care and in education, infrastructure, and welfare. In the latter case, I obtained the investment series for each parish before and after the establishment year and aggregated them to a health district level, which allowed us to carefully determine the intervention dates. In the analysis, to avoid purely administrative changes, I rely on the implementation dates accommodated with the public health investment series. All urbanised (*stad*) and semi-urbanised (*köping*) parishes are excluded and therefore the sample comprises only rural parishes (*land*). I also exclude from the analysis the rural parishes that were developed throughout the period into small towns (*köping*), parishes that experienced several health district re-allocations or those where the adoption dates were uncertain (220 out of 2353 parishes). Although the creation of the medical districts continued from 1890 up until the 1940s, I stop following the establishment of medical districts in 1917. Primarily, the organisation of districts after 1920 became largely administrative, when several medical units established a few decades prior were merged into a larger unit with no corresponding employment of medical practitioners. The public investment series at parish level are also unavailable for the period shortly after 1917. Additionally, the availability of medical personnel, such as midwives, began to stagnate as institutional childbirth deliveries increased gradually in rural locations. Finally, the waves of Spanish flu, which came to Sweden in 1918–1919, not only affected the subsequent cohorts but also encouraged the revision of the public control of infectious diseases.

In the individual administrative data, cohorts from 1890-1917 are not linked to their families of origin. To fill the gap in the individual's background characteristics, which is highlighted as necessary in early-life studies (Kuh and Ben-Shlomo 2007), I augment abundant parish-level information from other national records. The Swedish decennial censuses 1880-1910 are the main sources (Riksarkivet 2014). The counts contain the occupation names, their HISCO and status codes, which I further standardise into a historical international social class scheme and obtain a measure of socio-economic status consistent between the cohorts (HISCLASS; van Leeuwen and Maas 2011). Among the socio-economic variables at the parish level, I construct several, such as the share of elite and industrial workers, the share of agricultural workers, the share in the labour force and married in total aged 15-55, the mean family size, whether the parish had a railway or water supply installation. I supplement these variables with demographic characteristics of the parishes, such as the size of the population, the mean age of females, the share of females, the share of infants, the share of the population older than age 55 and the share of (non)disabled persons in the total population. Introducing population density, where area measures are calculated based on the historical maps (Riksarkivet 1890-1917), instead of the logarithm of the population, provides similar results. I complement this group of variables with information on deaths under age 15 gathered from the national death register (Sveriges Släktforskarförbund 2017).

Data for health districts are gathered from official statistical sources:

- o Statistiska Centralbyrån. (1880–1917). *BISOS U: Bidrag till Sveriges Officiella Statistik U. Kommunernas Fattigvård och Finanser [Communal Poor Relief and Finances]*. Stockholm: Norstedt & Söner [Annual Volumes].
- o Statistiska Centralbyrån. (1880–1910). *BISOS K: Bidrag till Sveriges Officiella Statistik K. Hälso- och Sjukvården [Health and Health Care]*. Stockholm: Norstedt & Söner [Annual Volumes].
- o Statistiska Centralbyrån. (1911–1917). *SOS: Sveriges Officiella Statistik. Allmän om Hälso- och Sjukvård [Health and Health Care]*. Stockholm: Norstedt & Söner [Annual Volumes].
- o Statistiska Centralbyrån. (1892). *BISOS R: Bidrag till Sveriges officiella statistik R. Valstatistik [Elections Statistics]*. Stockholm: Norstedt & Söner [Annual Volumes].
- o Riksarkivet. (1893–1946). *Medicinalstyrelsen: Årberättelse från Förste provinsialläkare i Malmohus län och Kristianstads län 1893–1946 [Annual Reports from First Provincial Doctors in Malmo and Kristianstad Counties]* [Annual Volumes].

Administrative divisions of health districts into parishes are gathered from the sources obtained in the state archives:

- o *Medicinalstyrelsen.* (1907). *Betänkande angående Rikets Indelning i Läkardistrikt samt Tjänstläkarnes Anställning och Åligganden [Divisions of the Country on Provincial Doctor Districts].* Stockholm: Norstedt & Söner.
- o *Medicinalstyrelsen.* (1939). *Riksarkivet. Rikets Indelning i Provinsialläkardistrikt före 1/7 1939 och Medicinalstyrelsens Yttrande och Förslag till Stadsläkarsakuniga den Maj 1932 [Divisions of the Country on Provincial Doctor Districts]*. Stockholm: Riksarkivet.

Information about specific initiatives related to the establishment of new health districts are gathered from the sources obtained in the national archives:

- o Medical History Database. (1885–1900). *Annual reports from the provincial doctors and local health boards 1885–1900.* Linköping University Electronic Press.
- o Riksarkivet. (1885–1914). *Årsberättelser från provinsialläkare. [Annual reports from the provincial doctors 1890-1914, all counties of Sweden]*.

Additional parish-level data are obtained from censuses and other sources:

- o Riksarkivet. (1880–1910). *National Sample of the 1880–1910 Census of Sweden [Decennial]*. Minneapolis: Minnesota Population Center [distributor].
- o Sveriges Släktforskarförbund. (1860–2016). *Sveriges Dödbok 1860–2016 [Swedish Death Book 1860–2016]*. Version 7.0. Stockholm: Sveriges Släktforskarförbund.

Parish-of-birth codes and names are based on the following:

- o Moritz, Sara. (2017). *Kommungränskonverterare [Converter of the Commune Codes]. Lund University. Resource document.* The program obtains information from *Riksarkivet (1600–2017), Sveriges Nationell Arkivdatabas topografiska databas [Topographic Database of the Swedish National Archives]*.

Appendix C

Table C.1 – Characteristics of the old and newly established provincial health districts

Characteristic	*Established before 1881*	*Established 1881–1917*
Number of health districts	163	124
Number of parishes per health district	14,1	6,9
Total population per health district	22,617	11,779
Number of midwives employed	–	4,4
Midwives employed per 10,000 population	–	5,6
Number of (extra or provincial) doctors employed per 10,000 population	–	1,9
Real investments into health care, *pre-reform*, 1900SEK	18,266	16,148
Real investments in health care per 1000 population, *pre-reform*, 1900SEK	715	1,611
Real investments in health care, *reform*, 1900SEK	–	19,267
Δ in real investments into health care, 1900SEK	–	3,119
Δ in real investments into health care per 1,000 population, 1900SEK	–	684
Real total public spending, *pre-reform*, 1900SEK	196,182	142,668
Real total public spending per 1,000 population, *pre-reform*, 1900SEK	6,792	11,638
Real total public spending, *reform*, 1900SEK	–	150,805
Δ in total public spending, 1900SEK	–	8,317
Δ in total public spending per 1,000 population, 1900SEK		1,272

Note: Parish- and health-district indicators are gathered from Statistiska Centralbyrån, BISOS U and K (1880–1917). *Pre-reform* investment data (*real health care investment* and *real total public spending*) are obtained for the year 1880 for control parishes and for one year prior to the establishment of a health district for treated parishes; aggregated at the level of a health district. *Reform* investment data (*real health care investment* and *real total public spending*) is not available for control parishes and are obtained for the first year of the reform implementation for treated parishes; aggregated at the level of a health district.

Appendix D

Survivors of Cohorts under Study

For cohorts born between 1890 and 1917, SIP contains all individuals residing in Sweden 1968–2012 and having family links (children or siblings) after 1930. The cohorts born between 1890 and 1917 appear in the SIP dataset consistently between the ages 78 and 95. Individuals are observed in Population and Housing Census 1960 for the first time, and from 1968 onwards followed in registers on an annual basis. I, therefore, do not observe the individuals who died or permanently migrated from Sweden prior to 1960, or those individuals who were childless and with no sibling born after 1930. In SIP, the data on the parish of birth (together with the county of birth) are given in text format, and I use an automatic procedure to match these names to parish names in the treatment health district dataset that further checks them manually. In the estimation sample, out of 492 parishes treated by the reform, the representatives of the 414 parishes are eventually observed. Table D.1 compares parishes observed in the individual-level data with initial sample. These parishes are in general similar across the range of socio-economic and health characteristics. The results tentatively indicate that observed parishes have a lower share of married individuals in 1880, although other measures of fertility and wealth are not statically significant to discern any systemic differences.

I gathered information on first-year survivors born in rural areas (live births minus infant deaths) of the cohorts born 1890–1917 from Statistiska Centralbyrån (1880-1910; 1911-1917), and further compared it with counts of individuals with rural places of birth available in SIP by cohort and those which have valid information on the parish of birth. There is an increasing fraction of individuals observed in the SIP dataset compared to a rather stable fraction of first-year survivors from rural places. This indicates that the individuals born 1890–1917 were either dying at an increasing rate between 1 and 77 years old (for the whole sample probably leading to overrepresentation of individuals with better outcomes) or a larger share of individuals were covered based on family links (for the whole sample probably leading to overrepresentation of individuals with worse outcomes). Cross-checked with the data for the number of infants in the treated parishes from decennial Censuses 1880–1910, the share of individuals observed to age 78, out of all first-year survivors, is 0.388. The share of survivors for the cohorts 1890–1917 for the whole of Sweden, for both rural and urban areas, is 0.478 (Human Mortality Database 2017).

In Figure D.1, I compare life expectancy at age 78 for Sweden as a whole, obtained from (Human Mortality Database 2017), and for the estimation sample. Both the level and development of life expectancy at age 78 are similar across the samples. Conditional on survival to age 78, the individuals in the estimation sample die at a mean age of 86–87. This characteristic of samples does not deviate from the actual life expectancy at age 78 for the same cohorts in Sweden in total, which is equal to 8.5 years and is similar for every cohort between the study and national samples (Human Mortality Database 2017). One should notice that the method applied in the paper compares the individuals born within the same year and across parishes of certain characteristics and hence should account for the above selection issues. As expected, the life expectancy at age 78 is somewhat higher among individuals observed in treated parishes compared to those in control parishes, for the latter averaged across untreated parishes *(ever implementers*) and untreated matched parishes (*never implementers*). Noteworthy, regarding both health and income outcomes and treatment variables, implemented and matched samples are similar.

It is obvious that I examined the group of individuals in their old age, between 78 and 95, where selective processes could cause some cohort differences to emerge. This methodological issue was

specifically addressed by recent studies of similar cohorts (Cutler and Lleras-Muney 2010; Zajacova and Burgard 2013) and most studies of long-term outcomes (Almond 2006; Bhalotra and Venkataramani 2013), and suggest that the main results might be underestimated. Because I have the data for the number of infants at the parish level for each cohort (from censuses) and the number of individuals who survived to age 78 (from SIP), I estimate the impact of the reform on the fraction of old-age survivors. The results show a significant increase in the fraction of survivors as a result of the reform at 5-7% of the mean (see Table 3 in the main text), suggesting both the beneficial instantaneous effects on infant survival and that the treatment effects are underestimated. Among the robustness checks, I further apply both a two-stage Heckman (1979) selection procedure and van den Berg and Drepper (2011) approach suggesting to fit a shared-frailty model to the left-truncated data to analyse whether a selection to survival affects the main results, and these procedures do not affect the main results.

References (to the sources not cited in the main text)

Almond, D. (2006). Is the 1918 influenza pandemic over? Long-term effects of in utero influenza exposure in the post-1940 US population. *Journal of Political Economy*, 114 (4), 672–712.

Cutler, D., & Lleras-Muney, A. (2010). The education gradient in old age disability. In D. Weil (Ed.), *Research Findings in the Economics of Ageing* (pp. 101–120). Chicago: The University of Chicago Press.

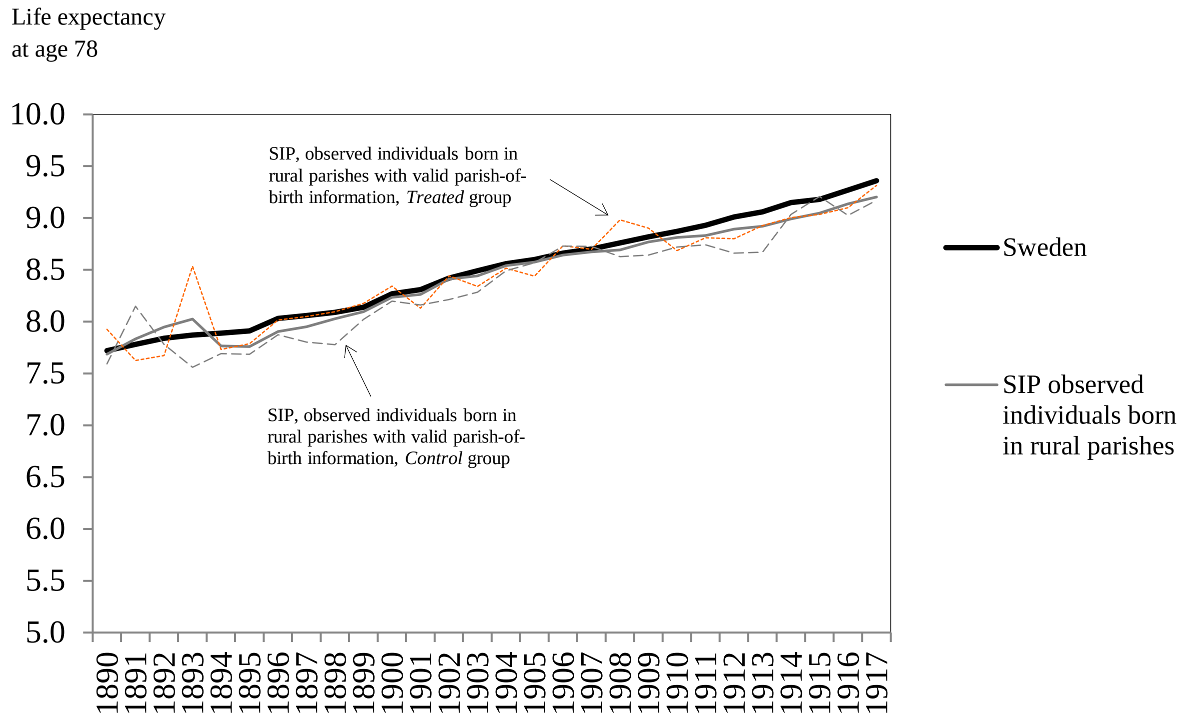

Figure D.1 – Life expectancy at age 78 in Sweden and estimation sample for the cohorts 1890–1917

Source: SIP and Human Mortality Database (2017)

Table D.1 – Pre-treatment differences between the parishes of birth observed and unobserved in the micro data, 1890–1917

	Observed=1	
	levels 1880	*differences 1890 to 1880*
	(1)	*(2)*
year of establishment	0.003	-
	(0.115)	
log real investment into health care per parish	0.003	-
	(0.903)	
log real education, infrastructure and welfare spending per parish	0.018	-
	(0.466)	
log population per parish	0.008	-0.137
	(0.705)	(0.309)
share of elite and industrial workers in male population 15–55 ages	-0.018	0.164
	(0.906)	(0.434)
share of agricultural workers in male population 15–55 ages	-0.175	0.026
	(0.274)	(0.891)
mean age of female	-0.002	-0.003
	(0.775)	(0.811)
share females in total	0.780	-1.174
	(0.412)	(0.290)
share in labour force in total 15–55 ages	-0.157	0.190
	(0.367)	(0.379)
share married in total 15–55 ages	-0.569*	1.017**
	(0.056)	(0.024)
mean family size	0.015	-0.036
	(0.675)	(0.505)
share under age 1 in total	-1.499	3.421
	(0.633)	(0.187)
share above age 55 in total	-0.616	0.667
	(0.362)	(0.400)
mortality rate under age 15	0.000	0.001
	(0.982)	(0.130)
share of (non)disabled	-0.598	-0.930
	(0.839)	(0.750)
railway	0.003	0.020
	(0.946)	(0.584)
water supplies improvements	0.107	0.019
	(0.224)	(0.844)
Parishes of birth	492	492

Note: OLS regression estimates. Among 492 parishes implemented the reform, the individuals born in 414 are observed in the individual data and 78 parishes are not. All characteristics are parish-level. Each coefficient is estimated separately. See Appendix B for data sources and descriptions. Parish-level indicators are gathered from Censuses 1880, 1890, 1900 and 1910, and from Statistiska Centralbyrån, BISOS U and K 1880–1917. Public investment data (*log real investment into health care per parish* and *log real education, infrastructure and welfare spending per parish*) are gathered for the year 1880 and reform years (varying across 1890–1917); due to the upward trend in all types of investments, differences between these investments correlate with a year of implementation by construction, therefore the results for these covariates are omitted.
P-values are in parentheses. *** p<0.01, ** p<0.05, * p<0.1

Appendix E

The Cause-of-Death Groups

The death and cause-of-death data were obtained from the Swedish death register and the Swedish cause-of-death register. These registers adopted the different revisions of the international classifications of the causes of death throughout 1968–2012, such as revision 8 for 1968–1986, revision 9 for 1987–1996, and revision 10 for 1997–2012. I classify all causes of death into five groups, such as infectious/respiratory diseases, cardiovascular diseases, diabetes, cancer, degenerative diseases of tissues and organs, and other causes including violent and ill-defined causes. Regarding the grouping of the causes of death among these revisions, the long-term follow-ups are reliable and valid (Janssen and Kunst 2004; Ludvigsson et al. 2011). Guided by the diagnostic groups suggested by the early-life epidemiological literature (e.g., Kuh and Ben-Shlomo 2007; Lynch and Davey Smith 2005). The exact codes used for these groupings are provided in the following table:

Table E.1 – Diagnosis groups across different revisions of the ICD, 1968–2012

	ICD-8	***ICD-9***	***ICD-10***
Infectious/respiratory diseases	000-136; 320-324; 460-519	001-139; 320-324; 460-519	A00-B99; G00-G09; J00-J99
Cardiovascular diseases	390-458	390-459	I00-I99
Diabetes	250	250	E10-E14
Cancer	140-239	140-239	C00-D48
Degenerative diseases	240-246; 251-315; 325-389; 520-789	240-246; 251-319; 325-389; 520-796	D50-E07; E15-F99; G10-H95; K00-R94
Other causes	790-796; E800-Y87	797-999; E800-V82	R95-Z99

References (to the sources not cited in the main text)

Janssen, F., & Kunst, A.E. (2004). ICD coding changes and discontinuities in trends in cause-specific mortality in six European countries, 1950-99. *Bulletin of the World Health Organization*, 82 (12), 904–913.

Ludvigsson, J.F., Andersson, E., Ekbom, A., Feychting, M., Kim, J., Reuterwall, C., et al. (2011). External review and validation of the Swedish national inpatient register. *BMC Public Health*, 11 (450), 1–16.

Appendix F

Matching Procedure

Figure F.1 plots a cumulative number of parishes by their dates of the reform implementation. 492 out of 2133 rural parishes established new health districts. From a sample of parishes untreated by the reform, I select one-to-one matches for each treated parish, based on the variety of the pre-treatment parish and health-district characteristics at both levels and trends. The motivation for the determinants of health district implementation is a standard model of public health care utilisation that involves socio-economic, infrastructure, health, demographic and health system domains (Andersen and Aday 1978; Kifmann 2005). The list of parish-level characteristics employed in the matching procedure is precisely the same as used among pre-treatment characteristics and includes their levels in 1880 and the differences between 1890 and 1880. In addition, I include health-district characteristics describing pre-treatment wealth and the public health system in the parishes, such as the logarithm of real investment in public health and the logarithm of real investment in education, infrastructure, and welfare.

For this approach, I calculate propensity scores and apply a nearest neighbour matching, in which I allow it to find only one control without replacement and impose a common support restriction, with a caliper 0.10. Narrowing or widening of the caliper gives qualitatively analogous results. After the matching, the control and treated groups exhibit a more similar distribution by propensity scores (see Figure F.2). As can be seen in Table F.1, the matching procedure results in 432 treated and 432 matched parishes. After the procedure, there are no significant differences between the treated and control groups of parishes by all parish- and health-district level characteristics. The majority of the parishes from the matched sample, which did not establish a health district and kept their names as for 1880-1906 (Medicinalstyrelsen 1907), or which got reformed after 1917 and were thus named as for 1939 (Medicinalstyrelsen 1939), could be linked to the individual parishes-of-birth records, for those born in 1890-1917. This linkage resulted in 660 parishes of birth with valid parish names that are further used in the analysis.

With a matching strategy, I could assign the reform treatment to years preceding/following the year of birth, instead of focusing on year of birth specifically. Thus, I could assign the reform treatment to fetal stage, and to older ages, and comparing children in prenatal period treated and untreated, at age 1 treated and untreated, at age 2 treated and untreated and so forth. Unlike for infancy, these analyses show that there are no effects of the reform on all-cause and CVD mortality for children treated by the reform in fetal stage or in ages 1–5.

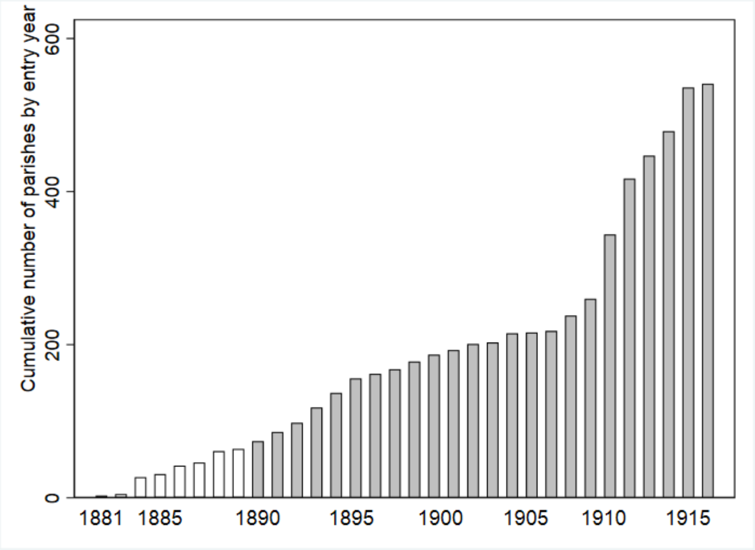

Figure F.1 – Number of parishes by their date of entry into treatment, 1881–1917

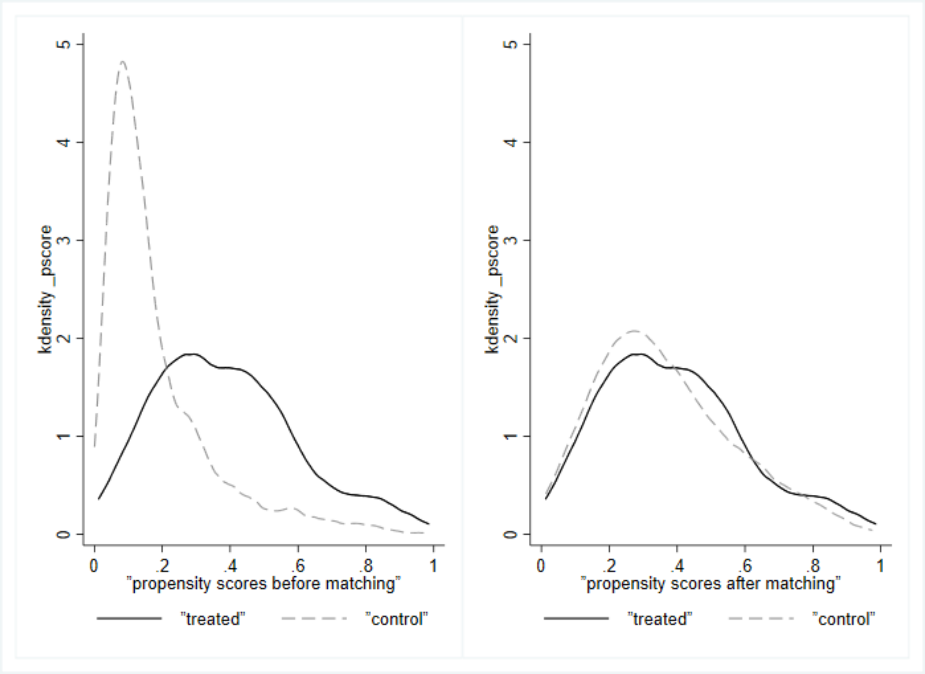

Figure F.2 – Density of the parishes of birth over the propensity scores

Table F.1 – Parish and health-district pre-treatment characteristics used for the full sample (control and implemented) and matched sample

	Before matching			*After matching*		
	Control	***Treated***		***Control***	***Treated***	
Variable	***Mean***	***Mean***	***p-value***	***Mean***	***Mean***	***p-value***
	1	***2***	***3***	***4***	***5***	***6***
Levels			1 versus 2			5 versus 6
log real health care investment per parish*	6.422	7.260	0.000	7.208	7.126	0.153
log real education, infrastructure and welfare spending per parish*	8.988	9.402	0.000	9.363	9.330	0.408
log total population 1880 per parish	7.130	7.232	0.011	7.277	7.226	0.335
share elite and industrial workers 1880	0.256	0.266	0.045	0.262	0.264	0.731
share agricultural workers 1880	0.421	0.406	0.005	0.410	0.408	0.867
mean age of female 1880	29.295	29.250	0.638	29.241	29.250	0.943
share females 1880	0.510	0.507	0.010	0.507	0.507	0.500
share in labour force 1880	0.674	0.666	0.074	0.666	0.666	0.996
share married 1880	0.480	0.480	0.800	0.483	0.482	0.859
share infants 1880	0.025	0.025	0.706	0.025	0.025	0.573
share older 55 ages 1880	0.150	0.150	0.670	0.150	0.150	0.883
under 15 mortality rate 1880	0.695	0.753	0.617	0.868	0.818	0.788
share (non)disabled 1880	0.992	0.993	0.024	0.992	0.992	0.560
mean family size 1880	4.037	4.015	0.316	4.030	4.025	0.890
railway1880	0.235	0.234	0.968	0.229	0.226	0.935
water supplies 1880	0.045	0.037	0.415	0.035	0.037	0.855
Differences						
diff log total population 1880-1890	-0.027	-0.027	0.792	-0.016	-0.020	0.657
diff share elite and industrial workers 1880-1890	-0.015	-0.009	0.093	-0.004	-0.008	0.408
diff share agricultural workers 1880-1890	0.021	0.013	0.106	0.010	0.014	0.547
diff mean age of female 1880-1890	1.140	1.319	0.017	1.262	1.202	0.561
diff share females 1880-1890	0.001	0.001	0.590	-0.001	0.001	0.312
diff share in labour force 1880-1890	0.023	0.028	0.136	0.028	0.028	0.985
diff share married 1880-1890	0.004	0.002	0.264	0.003	0.003	0.940
diff share infants 1880-1890	-0.001	-0.002	0.223	-0.002	-0.002	0.348
diff share older 55 ages 1880-1890	0.026	0.028	0.070	0.026	0.026	0.994
diff under 15 mortality rate 1880-1890	6.604	6.226	0.629	6.637	6.359	0.811
diff share (non)disabled 1880-1890	-0.001	-0.001	0.369	-0.001	-0.001	0.991
diff mean family size 1880-1890	-0.185	-0.189	0.806	-0.192	-0.183	0.656
diff railway 1880-1890	-0.090	-0.077	0.560	-0.069	-0.085	0.596
diff water supplies 1880-1890	0.010	0.016	0.555	0.019	0.016	0.847
Number of parishes	1641	492		432	432	
Number of health districts	163	107		131	96	

Note: *log real health care investment per parish and log real education, infrastructure and welfare spending per parish are obtained for year 1880 for control parishes and for one year prior to the establishment of a health district for treated parishes.

Table F.2 – Hazard ratios. Merging treatment to the ages beyond infancy. Long-term effect of the reform on mortality for ages 78–95, cohorts 1890–1917 Sweden. *Matched* sample.

	Fetal	***Age 1***	***Age 2***	***Age 3***	***Age 4***	***Age 5***
All-cause mortality						
post X new health district	1.011	0.981	1.021	1.012	1.011	1.003
p-value	(0.508)	(0.206)	(0.211)	(0.451)	(0.541)	(0.863)
Individuals	67,752	67,030	64,090	60,989	57,579	53,897
Deaths	62,401	61,856	59,190	56,415	53,288	49,990
Cardiovascular disease mortality						
post X new health district	1.027	0.983	1.024	1.020	1.030	1.025
p-value	(0.238)	(0.409)	(0.265)	(0.418)	(0.227)	(0.305)
Cohort FE	yes	Yes	yes	yes	yes	yes
Parish of birth FE	yes	Yes	yes	yes	yes	yes
Individuals	67,752	67,030	64,090	60,989	57,579	53,897
Deaths	35,125	35,374	33,990	32,648	31,033	29,393

Note: exponentiated coefficients from Cox stratified partial likelihood models. Models are adjusted for the left-truncation at age 78. Standard errors are clustered at the parish-of-birth level (660 parishes).
P-values are in parentheses. *** p<0.01, ** p<0.05, * p<0.1

References (to the sources not cited in the main text)

Andersen, R., & Aday, L. (1978). Access to medical care in the U.S. realized and potential. *Medical Care*, 16 (7), 533–346.

Appendix G

Table G.1 – Comparison of the 78–95 age group to younger age groups by demographic and socio-economic characteristics, Sweden cohorts 1890–1917

Characteristic	*Ages 50–66*	*Ages 67–77*	*Ages 78–95 (=included into analysis)*	*Difference in means p-value*	*Difference in means p-value*
1	*2*	*3*	*4*	*4 versus 2*	*4 versus 3*
Region of birth					
North	0.174	0.174	0.168	0.047	0.053
Centre	0.310	0.313	0.310	1.000	0.985
South	0.516	0.513	0.522	0.185	0.021
Sex					
Male	0.501	0.496	0.451	0.000	0.000
Female	0.499	0.504	0.549	0.000	0.000
Education					
Primary	0.511	0.466	0.469	0.000	0.946
More than primary	0.039	0.036	0.037	0.409	0.791
Unknown	0.450	0.498	0.494	0.000	0.469
Real mean annual income, 1000s (average over the age interval)	2.950	2.594	2.574	0.000	0.831
Sector of employment					
Agriculture	0.083	0.081	0.079	0.064	1.000
Industry	0.257	0.239	0.223	0.000	0.000
Service	0.251	0.237	0.244	0.025	0.103
Unknown	0.409	0.443	0.454	0.000	0.002
Marital status					
Married	0.409	0.405	0.418	0.000	0.000
Widowed	0.018	0.041	0.117	0.020	0.000
Divorced	0.002	0.003	0.005	0.000	0.000
Unknown	0.571	0.551	0.460	0.000	0.728
Death					
Alive	0.909	0.726	0.080	0.000	0.000
Ceased	0.091	0.274	0.920	0.000	0.000
Cause of death (among ceased)					
Infectious/Respiratory	0.036	0.057	0.091	0.000	0.000
CVD	0.437	0.564	0.569	0.000	0.000
Diabetes	0.012	0.014	0.018	0.000	0.000
Cancer	0.311	0.264	0.156	0.000	0.000
Degenerative	0.080	0.067	0.133	0.000	0.000
Accidents/Unknown	0.124	0.034	0.033	0.000	0.000

Note: OLS regression estimates. The significance of the differences in means is adjusted with the Bonferroni multiple-comparison test. The analysis is based on a sample of individuals born in parishes that ever implemented a reform: characteristics other than income – 54,959 individuals in ages 50–66, 54,544 individuals in ages 67–77, and 39,604 individuals in ages 78–95; income – 53,141 individuals in ages 50–66, 53,748 individuals in ages 67–77, and 38,618 individuals in ages 78–95. Individuals in ages 50–66 include cohorts 1902–1917, in ages 67–77 – cohorts 1891–1917.

Source: SIP

Appendix H

Additional Results for Mortality

In the paper, the specificities of the estimation sample preclude the use of particular estimators. As the study analyses old-age mortality in ages 78–95, I apply a Cox proportional hazard model that captures the non-linearity of the mortality rates and leaves the baseline hazards unspecified (Cox 1972). Across all specifications, tests based on Schoenfeld residuals reveal no violation in the proportionality of the hazards. For cause-specific mortality, Cox proportional hazards models are also applied. I estimate the effects for the mortality outcomes based on Eq.2 that eliminates the coefficients for the parishes of birth from the likelihood function, in analogy with parish-of-birth fixed effects (Allison 2011). They are estimated with the option of stratification (on parishes of birth) while running a standard Cox regression. As robustness checks, I estimate the Gompertz regression (Thatcher et al. 1998) presented in Table H.1 and linear probability models with death as an outcome for several age thresholds presented in Table H.2. All duration models adjust for left-truncation at age 78.

Table H.1 – Robustness analyses. Gompertz proportional hazards model. Effect of the reform on mortality in ages 78–95, cohorts 1890–1917

	I				***M***
	1	***2***	***3***	***4***	***5***
post X new health district	0.944**	0.945**	0.943*	0.959*	0.959**
	(0.016)	(0.019)	(0.060)	(0.088)	(0.025)
Cohort FE	yes	yes	yes	yes	yes
Parish of birth FE	yes	yes	yes	yes	yes
County of birth x cohort linear trends		yes			
Parish of birth *X*s x cohort FE			yes		
Parish of birth x cohort linear trends				yes	
Individuals	39,604	39,604	39,604	39,604	69,939
Deaths	36,429	36,429	36,429	36,429	64,451

Note: Models are adjusted for the left-truncation at age 78. *I* denotes a sample of implemented parishes of birth, *M* – sample of implemented and matched. See main text for further description. *Parish of birth Xs* denote parish-level pre-treatment control variables and include levels in 1880 and differences 1890–1880 of the following variables: log of total population, share of elite and industrial workers in male population 15–55 ages, share of agricultural workers in male population 15–55 ages, mean age of females, share of females in total population, share of population in labour force aged 15–55, share of married among population aged 15–55, share of infants in total population, share individuals older than 55 in total population, mortality rate under age 15, share of disabled in total population, mean family size, whether a parish had a railway, whether a parish had water installations. Standard errors clustered at the parish of birth level (414 parishes for *I*, 660 parishes for *M*).
P-values are in parentheses. *** p<0.01, ** p<0.05, * p<0.1

Table H.2 – Robustness analyses. Effect of the reform on alternative measures of survival in ages 78–95, cohorts 1890–1917

	I				***M***
	1	***2***	***3***	***4***	***5***
dead=1, OLS, 78–82 ages					
post X new health district	-0.0187**	-0.0205**	-0.0165	-0.0208**	-0.0119*
	(0.048)	(0.033)	(0.193)	(0.041)	(0.088)
dead=1, OLS, 78–87 ages					
post X new health district	-0.0239**	-0.0230**	-0.0255**	-0.0208*	-0.0205***
	(0.026)	(0.030)	(0.043)	(0.056)	(0.007)
dead=1, OLS, 78–95 ages					
post X new health district	-0.0019	-0.0017	-0.0074	-0.0015	-0.0034
	(0.717)	(0.751)	(0.313)	(0.804)	(0.431)
ln time alive, Tobit, 78–95 ages					
post X new health district	0.0769**	0.0761**	0.0572	0.0704***	0.0475**
	(0.014)	(0.017)	(0.128)	(0.000)	(0.034)
Cohort FE	yes	yes	yes	yes	yes
Parish of birth FE	yes	yes	yes	yes	yes
County of birth x cohort linear trends		yes			
Parish of birth *X*s x cohort FE			yes		
Parish of birth x cohort linear trends				yes	
Individuals	39,604	39,604	39,604	39,604	69,939

Note: *I* denotes a sample of implemented parishes of birth, *M* - sample of implemented and matched. See main text for further description. For the tobit models, the observations are right-censored at 2.890 (log of 18 years). Mean in not-treated is 9.47 years. See Table H.1 for the controls. Standard errors are clustered at the parish of birth level (414 parishes for *I*, 660 parishes for *M*).
P-values are in parentheses. *** p<0.01, ** p<0.05, * p<0.1

Appendix I

Sex-specific results

Table I.1 – Effect of the reform on cause-specific mortality by sex in ages 78–95, cohorts 1890–1917 Sweden

	All-cause	*Infectious/ Respiratory*	*Cardiovasc.*	*Diabetes*	*Cancer*	*Degener.*	*Other*
I							
post X new health district X men	0.929***	0.850**	0.931**	0.937	0.912	1.032	0.976
p-value	(0.003)	(0.040)	(0.048)	(0.702)	(0.106)	(0.655)	(0.872)
post X new health district X women	0.950**	0.956	0.945*	0.952	0.933	0.986	0.894
p-value	(0.043)	(0.604)	(0.063)	(0.766)	(0.257)	(0.824)	(0.440)
Cohort FE	yes	yes	yes	yes	yes	yes	yes
Parish of birth FE	yes	yes	yes	yes	yes	yes	yes
Individuals	39,604	39,604	39,604	39,604	39,604	39,604	39,604
Deaths	36,429	3,304	20,733	649	5,675	4,847	1,221
M							
post X new health district X male	0.934***	0.910	0.933**	1.004	0.885***	1.004	1.096
p-value	(0.000)	(0.122)	(0.013)	(0.976)	(0.007)	(0.943)	(0.402)
post X new health district X female	0.981	0.996	0.971	0.972	0.922*	1.043	1.072
p-value	(0.305)	(0.950)	(0.234)	(0.816)	(0.089)	(0.411)	(0.519)
Cohort FE	yes	yes	yes	yes	yes	yes	yes
Parish of birth FE	yes	yes	yes	yes	yes	yes	yes
Individuals	69,939	69,939	69,939	69,939	69,939	69,939	69,939
Deaths	64,451	5,862	36,579	1,185	9,961	8,723	2,141

Note: exponentiated coefficients from Cox stratified partial likelihood models (hazard ratios). Models are adjusted for the left-truncation at age 78. *I* denotes a sample of implemented parishes of birth, *M* – sample of implemented and matched. Standard errors are clustered at the parish of birth level (414 parishes for *I*, 660 parishes for *M*). See main text for further description. P-values are in parentheses. *** p<0.01, ** p<0.05, * p<0.1

Table I.2 – Effect of the reform on ln income by sex for ages 78–95, cohorts 1890–1917 Sweden

	I	*M*
post X new health district X male	0.0249	0.0121
p-value	(0.183)	(0.380)
post X new health district X female	0.0164	0.0211**
p-value	(0.136)	(0.019)
Individuals	38,618	68,224
R-sq	0.241	0.288

Note: Linear fixed-effects regression estimates. *I* denotes a sample of implemented parishes of birth, *M* – sample of implemented and matched. Standard errors are clustered at the parish of birth level (414 parishes for *I*, 660 parishes for *M*). See main text for further description.
P-values are in parentheses. *** p<0.01, ** p<0.05, * p<0.1

Appendix J

Additional Results for ln Income

Regarding the long-term income, to provide a comparison between the cohorts with different earnings profiles and to impede their disproportionate influence on the results, the logarithm of income is used as an outcome in linear least squares models. In addition to the average of the real income in ages 78–95 that is the preferred outcome measure, I run the models with shorter age intervals and with the average of the residual incomes (*life-time earnings*), which is argued to reduce the variation from measurement error (Lindahl et al. 2015). *Life-time earnings 78-95 ages* are defined in two stages: first, log real labour income for each individual-year (in a sample between the age of 78 and a year prior year of death or the age of 95) is regressed on *year-of-birth* linear and squared terms and *year* dummies and residual for each individual-year is estimated; second, mean of the residual for each individual, that is *life-time earnings in 78–95 ages*, is calculated. Using linear and squared terms of *age* instead of *year-of-birth* produces similar results. Please see Table J.1 for these and additional results.

References (to the sources not cited in the main text)

Lindahl, M., Palme, M., Sandgren Massih, S., & Sjogren, A. (2015). Long-term intergenerational persistence of human capital: An empirical analysis of four generations. *Journal of Human Resources*, 50 (1), 1–33.

Table J.1 – Robustness analyses. Long-term effects of the reform on ln income, cohorts 1890–1917

	I				***M***
	1	***2***	***3***	***4***	***5***
life-time earnings 78–95 ages					
post X new health district	0.0199**	0.0217**	0.0196**	0.0238**	0.0204***
	(0.037)	(0.032)	(0.045)	(0.027)	(0.007)
Individuals	38,618	38,618	38,618	38,618	68,224
ln income 78–82 ages					
post X new health district	0.0222**	0.0246**	0.0261*	0.0236**	0.0182**
	(0.028)	(0.024)	(0.019)	(0.041)	(0.023)
Individuals	38,609	38,609	38,609	38,609	68,208
ln income 78–87 ages					
post X new health district	0.0208**	0.0214**	0.0227**	0.0245**	0.0169**
	(0.035)	(0.040)	(0.029)	(0.034)	(0.027)
Individuals	38,615	38,615	38,615	38,615	68,218
ln income 78–95 ages, closest value for income instead of the null					
post X new health district	0.0156*	0.0161*	0.0218**	0.0210**	0.0143**
	(0.066)	(0.074)	(0.017)	(0.040)	(0.036)
Individuals	38,618	38,618	38,619	38,619	68,224
ln income 78–95 ages, Excluding years under widowhood					
post X new health district	0.0225*	0.0241*	0.0261*	0.0307**	0.0233**
	(0.086)	(0.080)	(0.065)	(0.045)	(0.016)
Individuals	27,824	27,824	27,824	27,824	48,941
ln income 78–95 ages, Excluding cohorts 1890–1892					
post X new health district	0.0186**	0.0169*	0.0193**	0.0181*	0.0131*
p-value	(0.047)	(0.082)	(0.047)	(0.072)	(0.068)
Individuals	38,173	38,173	38,173	38,173	67,551
Cohort FE	yes	yes	yes	yes	yes
Parish of birth FE	yes	yes	yes	yes	yes
County of birth x cohort linear trends		yes			
Parish of birth *X*s x cohort FE			yes		
Parish of birth x cohort linear trends				yes	

Note: Linear fixed-effects regression estimates. *Parish of birth Xs* denote parish-level pre-treatment control variables and include levels in 1880 and differences 1890–1880 of the following variables: log of total population, share of elite and industrial workers in male population 15–55 ages, share of agricultural workers in male population 15–55 ages, mean age of females, share of females in total population, share of population in labour force aged 15–55, share of married among population aged 15–55, share of infants in total population, share individuals older than 55 in total population, mortality rate under age 15, share of disabled in total population, mean family size, whether a parish had a railway, whether a parish had water installations. See main text for further description. Standard errors are clustered at the parish of birth level.
P-values are in parentheses. *** p<0.01, ** p<0.05, * p<0.1

Appendix K

Robustness Analyses (test results are presented in the main text)

This Appendix contains a detailed description of the robustness tests and their results.

Due to the gradual implementation of the reform throughout Sweden in 1890–1917, the study applies a DiD approach. Given the numerous changes in demographic and economic conditions in the late-19th and early 20th centuries, a DiD method applied in the paper has several advantages. The smooth period changes, potentially affecting childhood conditions such as a rise in real wages, mortality decline or decline in fertility rates, in different locations are ruled out by the introduction of the year of birth fixed effects. The parish-specific differences invariant over time, such as the local wealth, climate or institutions, are also controlled by inclusion of parish-specific dummies. As the health district was introduced to a group of parishes, it is possible to include group fixed effects instead, although I settled for the parish fixed effects as more demanding. In all models, to account for the location-level unobserved correlation, standard errors are clustered by the parish of birth. All models additionally introduce sex dummies to control for the sex-specific differences in survival and income trajectories.

While in the main analysis, I limit the sample to children born 5 years before and after the reform mainly because the state guaranteed the placement of a doctor for 5 years, in case the doctor and the initiatives were in place afterward, the results should hold if I include more cohorts. I thus estimate the models while including individuals 7, 10 and 14 years before and after the reform (see Table K.1). In general, the results stay qualitatively similar to the main results. Note that the results for 7 and 10 years for ln income for individuals in the implemented sample somewhat decrease in size and become statistically insignificant, albeit not for the matched sample. This can be explained by the fact that the implemented sample now contains individuals aged 7–10 in the control group which might have been affected by the increases in investment into primary schooling (*folkskola*) or beneficial changes from the establishment of health districts for the pupils, such as closures of schools during epidemics outbreaks. In support of this, as soon as I control for the pre-treatment trends in district-level investment into schooling, infrastructure and poor relief (*investmentXcohort dummies*), the coefficient for *postXhealth-district* increases up to 0.0220 (p-value 0.039) for a 7-year period, and to 0.0189 (p-value 0.073) for a 10-year period. The effects for the age group 0–14 are in line with the main results of the paper.

The main results are robust to observable and unobservable pre-treatment differences in treatment groups, and I have already included a variety of factors deemed important for the earlier adoption of the reform. Nevertheless, I collected more information on the local leadership. As argued by Carlsson (1987), a number of residents having voting rights (including women) and less than 1/50 of parish income and wealth were observed to influence the adoption of public goods at the local level in Sweden. Based on official statistical sources (Statistiska Centralbyrån 1892), I collect this variable at parish- and health-district levels. As expected, a larger share of middle-class residents with voting rights is significantly and positively associated with earlier reform adoption. However, adding this variable into the specification, as an interaction with cohort dummies, does not affect the main results (see in the main text Table 8 Panel B). In the matching procedure, voting rights per capita are not significant in explaining the treatment, implying that the factors that are already considered in the model approximate the influence of local leadership.

An additional concern relates to selective mortality and its potential effects on the main results. This methodological issue was specifically addressed by recent studies of similar cohorts (Cutler and Lleras-Muney 2010; Zajacova and Burgard 2013) and most studies of long-term outcomes (Almond 2006;

Bhalotra and Venkataramani 2013), and suggest that the main results might be underestimated. Before I estimate the impact of the reform on the fraction of old-age survivors, I further apply a two-stage Heckman correction procedure to analyse whether a selection to survival affects the main results (Heckman 1979). For Panel C Table 8 in the main text (Heckman two-stage procedure), in the first stage, the probability of being observed in the estimation sample is modelled as a function of cohort fixed effects, county of birth fixed effects and sex for all individuals whom I observe in the year 1960 (*1960 Census*) in a probit model. An inverse Mills' ratio originating for each individual from the estimates of the probit model is further included as a covariate into the baseline specification. As can be seen, this procedure does not affect the main results. One more approach to correct for survival bias potentially affecting the results of this study is van den Berg and Drepper (2011) approach. It suggests fitting a shared-frailty model to the left-truncated data, which is relevant in our case, when certain parishes may systematically have shared reasons for appearing or not appearing due to, for example, selective migration, in the estimation sample (ages 78–95). The exponentiated point estimate in Panel D Table 8 in the main text is smaller in comparison to the baseline one, although is not statistically different when this approach is applied. This is line with an indication by van den Berg and Drepper (2011): in case of dynamic selection and left-truncation, models that are not properly accounting for these data generating processes produce the coefficient estimates biased toward zero. Noteworthy, the medical treatments for certain diseases, such as antibiotics and drugs for cardiovascular and heart diseases and diabetes, which emerged as contemporaneous events, should not affect the main results. Finally, it can be also questioned whether a mean income in old age can approximate lifetime income, as pension and capital income only partially rely on the economic performance in adulthood. While I believe that the use of income in old age as lifetime income leads to the underestimation of the treatment effects, it can also be viewed a measure of an individual's economic well-being (Netuveli et al. 2006).

In the period under analysis, it is possible that some abrupt changes affected parishes differently, thus potentially harming the identification strategy. One potential threat to the estimates arises from the mass emigration of Swedish residents to the US and other countries, which was both discontinuous and region-specific in the period in question. If the emigrating population was selective towards poorer socio-economic classes, thus affecting families of the cohorts under study, and occurred in a sharp manner across different regions (coinciding with conditions that lead to the adoption of the health district reform), one might expect a disturbance in the estimates. The emigration to other countries from Sweden was massive between 1880 and 1910, and approximately 80% of all migrants left for the US (Statistiska Centralbyrån 1969). Regarding the age composition of the migrants, ages 15 to 29 predominated. The primary emigrant counties, where the countryside experienced mass migration to the US, were Värmland and Halland (Bohlin and Eurenius 2010). I perform several robustness checks to account for the potential effects from the selective emigration to the US. Panel E of Table 8 in the main text presents the results for the sample, where I exclude the individuals from the counties of birth mostly affected by emigration to the US. As seen, the main results are unaffected by this exclusion. Next, to account for both unfavourable mortality and economic conditions in the parishes, I include additional interaction terms into the models, (parish and year-specific) *under-15-mortality rateXshare of skilledXyear-of-birth* dummies (the underlying two-way interactions and main effects are also included). Panel F in Table 8 in the main text shows estimates that are not different from the baseline ones. These results are similar if to use interactions with pre-treatment levels of *under-15-mortality rateXshare of skilled* instead. Finally, I perform a bounds exercise, dropping parishes at different top percentiles of the distribution of the SES (HISCLASS) variable obtained from decennial censuses. Data from official yearbooks (Statistiska Centralbyrån 1890, 1900, 1910) allows me to distinguish the county-level migrant rate for the poorest strata – impoverished rural workers and domestic servants. Increasing this rate by certain thresholds could give an indication of not only how many poor families emigrated spatially, but also

how they responded to the improving employment opportunities in 1890–1917 in Sweden compared to the US. In Table K.2, for both mortality and ln income, I drop individuals in the treated parishes at the subsequent top percentiles (equivalent to the percentage of poor emigrants increased by 30%, 2 times, 5 times, 10 times, 20 times, 50 times, and 100 times) of the distribution of the SES (HISCLASS) variable. It is equivalent to dropping the wealthiest parishes of birth, which probably lost the largest share of the poorest due to emigration. At the few bounds of the exercise, no parishes are dropped, because neither of them belongs to the top percentile of the SES distribution. This exercise shows that the estimated effects are fairly stable and statistically significant in all subsamples. I stop this exercise at a 100 times increase in the share of the poor US emigrants, which is a rather extreme number for the context under analysis. Based on the empirical analysis by Bohlin and Eurenius (2010), a response of the migrant rate to the improving wages in Sweden compared to the US was not larger than 28%.

Furthermore, the location-specific influence of World War I might be important for the estimates. Even though Sweden was neutral during the war, the treated parishes could be those mostly affected by its threat, forcing the parents to postpone fertility until peaceful times, or those agricultural regions that witnessed an increase in the exports of the raw materials to Europe, thereby boosting the local wages (Qvarnström 2014; Siney 1975). I, therefore, run the analysis omitting from the sample individuals born in Norrbotten and Västerbotten counties, those most affected by the war, and present the estimates in Panel G Table 8 in the main text. Again, both long-term income and mortality effects attain statistical significance and size analogous to the baseline coefficients. To supplement these analyses, I add to the models the parish of birth characteristics that more carefully describe the age structure of the population and vary across cohorts, in addition to those included previously. As Panel H Table 8 in the main text shows, the main results are also not sensitive to this check.

An additional robustness analyses have been performed with regard to whether the parents responded to the reform as such. It might be possible that families, hoping to improve the life chances of their new-borns, moved to areas with access to health care and had some advantageous characteristics that could instead explain the results. The internal migration flows in rural areas in the period under analysis are explained by the structure of the local labour force (Enflo et al. 2014). Previously, I found that the implementation of health care reform did not correlate with shares of industrial or agricultural workers in total or the share of married persons in total. To account for residential selection, I repeat the analysis by additionally controlling for several time-varying indicators of the local labour markets, such as the share of industrial and agricultural workers in the total male population aged 15–55, the share of skilled workers in the total male population aged 15–55, the share of the married in the total population aged 15–55, the mean age of females, and the share of the population of non-Swedish origin. Panel I Table 8 in the main text reports the results from the models including these controls, which appear to be unaffected compared to the baseline estimates. I additionally analyse the reform-driven migration responses by using the parish-level information on migrant structure obtained from censuses dated 1880–1910 (see Table K.3, Model 1). The results indicate that there are no effects of the reform on migration flow between the parishes. Finally, it is plausible that the reform generated fertility responses favouring the delivery of healthy new-borns. Again, I can test this with parish-level data from censuses (see Table K.3, Model 2); the results reveal no presence of these responses.

One could also be curious as to what extent the Spanish flu affected the main results. The Spanish flu arrived in 1918–1919 and therefore affected the health and income trajectories after the treatment by health care considered in this study, in childhood and prime working ages of the individuals. Therefore, this death and morbidity shock does not lead to selection bias in the treatment estimates, which instead demonstrate the total effects of the reform on income and mortality, but it could mediate these effects. Previous studies of Sweden found strong immediate effects of the Spanish flu pandemic on mortality (Åman 1990), but no effects on earnings (Karlsson et al. 2014). The previous literature is also consistent

in that the lasting health effects from the influenza pandemic emerge only for individuals exposed in utero (Bengtsson and Helgertz 2015). In the analysis, I run the models while excluding the individuals residing in the counties mostly hit by the Spanish flu: northern counties Koppaberg, Gävleborg, Västernorrland, Jämtland, Västerbotten and Norrbotten (Engberg 2009). As Panel J in Table 8 in the main text shows, the estimates for both mortality and income are not affected by their exclusion.

References (to the sources not cited in the main text)

Almond, D. (2006). Is the 1918 influenza pandemic over? Long-term effects of in utero influenza exposure in the post-1940 US population. *Journal of Political Economy*, 114 (4), 672–712.

Åman, M. (1990). *Spanska Sjukan: Den Svenska Epidemin 1918–1920 och Dess Internationella Bakgrund [Spanish Flu: The Swedish Epidemic 1918–1920 and Its International Background]*. Uppsala: Almqvist & Wiksell International Stockholm.

Bengtsson, T, & Helgertz, J, (2015). The long lasting influenza: The impact of fetal stress during the 1918 influenza pandemic on socioeconomic attainment and health in Sweden 1968–2012. *IZA Working Papers* 9327.

Bohlin, J., & Eurenius, A. (2010). Why they moved — emigration from the Swedish countryside to the United States, 1881–1910. *Explorations in Economic History,* 47 (4), 533–551.

Bütikofer, A., Løken, K.V., & Salvanes, K.G. (2015). Long-term consequences of access to well-child visits. *IZA Working Papers* 9546.

Carlsson, S. (1987). From four estates to two chambers: The Riksdag in a period of transition, 1809–1921. In M.F. Metcalf & H. Schück (Eds.), *The Riksdag: A History of the Swedish Parliament* (pp. 165–222). New York: St. Martin's Press.

Enflo, K., Lundh, C., & Prado, S. (2014). The role of migration in regional wage convergence: Evidence from Sweden 1860–1940. *Explorations in Economic History,* 52, 93–110.

Engberg, E. (2009). The invisible pandemic: Community response to pandemic influenza in rural northern Sweden 1918–20. *Varia Historia,* 25 (42), 429–456.

Karlsson, M., Nilsson, T., & Pichler, S. (2014). The impact of the 1918 Spanish flu epidemic on economic performance in Sweden. An investigation into the consequences of an extraordinary mortality shock. *Journal of Health Economics,* 36, 1–19.

Netuveli, G., Wiggins, R.D., Hildon, Z., Montgomery, S.M., & Blane, D. (2006). Quality of life at older ages: Evidence from the English longitudinal study of aging (wave 1). *Journal of Epidemiology and Community Health,* 60 (4), 357–363.

Qvarnström, S. (2014). "Sweden." In *1914-1918-Online: International Encyclopedia of the First World War*. Berlin: Freie Universität Berlin.

Siney, M. (1975). Swedish neutrality and economic warfare in World War I. *Conspects of History,* 1 (2), 13–28.

Statistiska Centralbyrån. (1892). *BISOS R: Bidrag till Sveriges Officiella Statistik R. Valstatistik. [Sweden's Official Statistics. Election Statistics]*. Stockholm: P.A. Norstedt & Söner.

Statistiska Centralbyrån. (1969). *Historisk Statistik för Sverige. Del 1. Befolkning 1720–1967 [Historical Statistics for Sweden. Part 1. Population 1720–1967]*. Stockholm: KL Beckmans Tryckerier AB.

Table K.1 – All-cause mortality and income outcomes. Effect of the reform with pre- and post-treatment periods 7, 10 and 14 years each, ages 78–95 cohorts 1890–1917 Sweden

	I				*M*
	1	***2***	***3***	***4***	***5***
All-cause mortality					
7 years					
post X new health district	0.937***	0.944***	0.919***	0.959*	0.947***
p-value	(0.001)	(0.004)	(0.001)	(0.060)	(0.001)
Individuals	49,731	49,731	49,731	49,731	84,417
Deaths	45,812	45,812	45,812	45,812	77,851
10 years					
post X new health district	0.945***	0.951***	0.921***	0.961*	0.956***
p-value	(0.001)	(0.004)	(0.000)	(0.050)	(0.002)
Individuals	55,976	55,976	55,976	55,976	93,845
Deaths	51,598	51,598	51,598	51,598	86,585
14 years					
post X new health district	0.954***	0.955***	0.934***	0.959**	0.964***
p-value	(0.003)	(0.006)	(0.000)	(0.035)	(0.006)
Individuals	61,459	61,459	61,459	61,459	102,886
Deaths	56,648	56,648	56,648	56,648	94,917
Ln income					
7 years					
post X new health district	0.0144	0.0133	0.0100	0.0134	0.0159**
p-value	(0.152)	(0.210)	(0.317)	(0.252)	(0.035)
Individuals	49,704	49,704	49,704	49,704	82,334
10 years					
post X new health district	0.0107	0.0083	0.0063	0.0100	0.0147**
p-value	(0.272)	(0.400)	(0.495)	(0.405)	(0.044)
Individuals	55,946	55,946	55,946	55,946	91,516
14 years					
post X new health district	0.0224**	0.0210**	0.0226***	0.0260**	0.0131**
p-value	(0.011)	(0.020)	(0.005)	(0.019)	(0.048)
Individuals	59,905	59,905	59,905	59,905	100,314
Cohort FE	yes	yes	yes	yes	yes
Parish of birth FE	yes	yes	yes	yes	yes
County of birth x cohort linear trends		yes			
Parish of birth Xs x cohort FE			yes		
Parish of birth x cohort linear trends				yes	

Note: exponentiated coefficients from Cox stratified likelihood models for all-cause mortality (hazard ratios), adjusted for the left-truncation at age 78, and linear fixed-effects regression estimates for ln income. *I* denotes a sample of implemented parishes of birth, *M* – sample of implemented and matched. *Parish of birth Xs* denote parish-level pre-treatment control variables and include levels in 1880 and differences 1890–1880 in the following variables: log of total population, share of elite and industrial workers in male population aged 15–55, share of agricultural workers in male population aged 15–55, mean age of females, share of females in total population, share of population in labour force aged 15–55, share of married among population aged 15–55, share of infants in total population, share of individuals older than 55 in total population, mortality rate under age 15, share of disabled in total population, mean family size, whether a parish had a railway, whether a parish had water installations. See main text for further description. Standard errors clustered at the parish of birth level.
P-values in parentheses. *** p<0.01, ** p<0.05, * p<0.1

Table K.2 – All-cause mortality and income outcomes. Robustness analysis with bounds, accounting for emigration of the poor to the US, ages 78–95 cohorts 1890–1917 Sweden

	Dropping the top percentiles equal to % of the poor emigrants increased by rate:						
	30%	***2 times***	***5 times***	***10 times***	***20 times***	***50 times***	***100 times***
All-cause mortality							
Dropping among the treated							
I							
post X new health district	0.941***	0.941***	0.940***	0.941***	0.942***	0.941***	0.939***
p-value	(0.006)	(0.006)	(0.006)	(0.007)	(0.009)	(0.008)	(0.006)
Parishes	414	414	413	412	411	411	409
Individuals	39,604	39,604	39,591	39,564	39,298	39,148	38,588
Deaths	36,429	36,429	36,418	36,394	36,145	36,010	35,500
M							
post X new health district	0.959**	0.959**	0.959**	0.960**	0.960**	0.961**	0.958**
p-value	(0.016)	(0.016)	(0.016)	(0.020)	(0.020)	(0.025)	(0.018)
Parishes	660	660	659	658	657	657	656
Individuals	69,776	69,776	69,763	69,677	69,504	68,360	66,549
Deaths	64,297	64,297	64,286	64,210	64,047	62,992	61,334
Ln income							
Dropping among the treated							
I							
post X new health district	0.0203**	0.0203**	0.0202**	0.0203**	0.0213**	0.0206**	0.0191*
p-value	(0.041)	(0.041)	(0.041)	(0.040)	(0.034)	(0.039)	(0.057)
parishes	414	414	413	412	411	410	408
Individuals	38,618	38,618	38,605	38,578	38,321	38,173	37,637
M							
post X new health district	0.0167**	0.0167**	0.0167**	0.0164**	0.0173**	0.0172**	0.0166**
p-value	(0.028)	(0.028)	(0.029)	(0.031)	(0.026)	(0.022)	(0.028)
Parishes	660	660	659	658	657	656	655
Individuals	68,064	68,064	68,051	67,967	67,802	66,680	64,927

Note: exponentiated coefficients from Cox stratified likelihood models for all-cause mortality (hazard ratios), adjusted for the left-truncation at age 78, and linear fixed-effects regression estimates for ln income. *I* denotes a sample of implemented parishes of birth, *M* – sample of implemented and matched. *% of the poor emigrants* are county- and year-specific. See main text for further description. Standard errors clustered at the parish of birth.
P-values in parentheses. *** p<0.01, ** p<0.05, * p<0.1

Table K.3 – Robustness analyses. Effect of the reform on migrant structure and household size of the parishes of birth, 1890–1917

	(1)<br>***Share of migrants***	*(2)*<br>***Household size***
post X new health district	0.00100	0.00141
	(0.372)	(0.851)
Cohort FE	yes	yes
Parish of birth FE	yes	yes
Observations (parish-of-birthXcohort)	3,278	3,278
R-squared	0.082	0.006

Note: Linear fixed-effects regression estimates. The analyses are restricted to parish-cohorts used in the sample. Data are obtained from Censuses 1880–1910 and recalculated to parish-of-birthXcohort level. Standard errors clustered at the parish of birth (414 parishes).
P-values in parentheses. *** p<0.01, ** p<0.05, * p<0.1

Appendix L

Comparison with other studies

To the best of my knowledge, this study is the first to find effects of the rural health policy on both individual health and income in oldest-old age. Due to the reform, individuals treated in the year of birth attain decreases in all-cause mortality risk at approximately 4.2-6.0%, roughly equivalent to 0.5-0.7 additional years spent alive. The positive effects on the total income amount to 1.6-2.5%.

I can briefly align the findings of policy-based studies focusing on age and cohort groups similar to or overlapping with this study. To date, the only policy-based study that discloses the effects for the oldest-old ages is Aizer et al. (2016). Based on the cash transfer programme initiated in the US in 1911-1935, with no public health components, scholars estimated the treatment effects on individuals accepted to the programme below age 18 and observed at ages 80 and older are approximately 1.4 additional years. This program also enhanced incomes in young adulthood by 13.6%. They study poor families and estimate an average treatment effect, so the effect on survival should be at least compared with that for the low SES in the present study, which equals to 0.6-1.1 years (which is still an intention-to-treat effect and thus of lower bound). Other policy-based studies looked at outcomes at younger ages. Bhalotra et al. (2016) and Bhalotra et al. (2017) have studied the long-term health effects of the infant care programme, implemented in 1931-1933 Sweden, and demonstrated a 7.0% reduction in mortality in ages 40-75 and increase in total incomes by 7.3% in ages 30s. Hjort et al. (2017) find that Danish cohorts treated in infancy by a similar program are 0.2–0.7% less likely to survive between ages 50–64, and are less likely to be diagnosed with cardiovascular disease, with no effects on earnings.

Other studies looking at old and oldest-old ages use negative exposures as quasi-experiments, such as disease outbreaks. For instance, Schellekens and van Poppel (2016) show that in the Netherlands a declining trend in infant mortality at birth and increase in height contributed to 3–5 years to rise in life expectancy at age 30 for cohorts born in 1812–1921. These effects appear to be larger than in the present study, although scholars pick up the effects for the younger ages and do not control for the unobserved factors plausibly driving the trends. The study, which is the most compatible with the present one in terms of age span and the cohorts, is Myrskylä et al. (2013), that follows the cohorts exposed to the Spanish influenza pandemic in 1918–1920 in the US between ages 63–95. Scholars find that those exposed at late gestation and at birth experience 8% higher mortality risks compared to the later born cohorts. For cohorts born in rural parishes of Scania in 1760–1895, Bengtsson and Lindström (2003) examine whether the individual's mortality risks in ages 55–80 are influenced by a transitory component in regional infant mortality rate in year of birth and food prices in a year prior to birth and a year of birth. Their results suggest that diminishing infant mortality at birth to its levels in the late 1910s, by the end of the treatment period in our study, would decrease later mortality by around 7%.

Broadly speaking, the effects on health and income obtained in the present study are sizable and stay within the scope of those demonstrated in the previous quasi-experimental studies.

References (to the sources not cited in the main text)

Bhalotra, S., Karlsson, M., Nilsson, T., & Schwarz, N. (2016). Infant health, cognitive performance and earnings: Evidence from inception of the welfare state in Sweden. *IZA Working Papers* 10339.
